# The real-world analysis of adverse events with azacitidine: a pharmacovigilance study based on the FAERS and WHO-VigiAccess databases

**DOI:** 10.3389/fphar.2025.1555838

**Published:** 2025-03-19

**Authors:** Zhaorui Wang, Linlin Guo, Youfu He, Baiquan Zhang, Yang Wang, Juan Ding

**Affiliations:** ^1^ Translational Medicine Research Center, The Fifth Clinical Medical College of Henan University of Chinese Medicine (Zhengzhou People’s Hospital), Zhengzhou, China; ^2^ The Second Department of Radiotherapy, The First Affiliated Hospital of Zhengzhou University, Zhengzhou, China; ^3^ Department of Cardiology, Guizhou Provincial People’s Hospital, Guiyang, China; ^4^ Department of Respiratory Medicine, The First Affiliated Hospital of Zhengzhou University, Zhengzhou, China; ^5^ AI & Data Innovations, Cluster BI Inc., Toronto, ON, Canada; ^6^ Department of Nursing, The First Affiliated Hospital of Zhengzhou University, Zhengzhou, China

**Keywords:** azacitidine, DNA methyltransferase inhibitor, FAERS, WHO-VigiAccess, disproportionality analysis, adverse events

## Abstract

**Background:**

Azacitidine is used to treat myelodysplastic syndrome (MDS) and acute myeloid leukemia (AML). It acts as a cytosine analog and DNA methyltransferase inhibitor, inducing DNA hypomethylation to reverse epigenetic modifications and restore normal gene expression. However, adverse events (AEs) associated with azacitidine are mainly reported in clinical trials, with limited real-world evidence. This study aims to assess the AE profile of azacitidine by utilizing data from the Food and Drug Administration (FDA) Adverse Event Reporting System (FAERS) and WHO-VigiAccess databases.

**Methods:**

We extracted adverse event (AE) reports related to azacitidine from the FAERS and WHO-VigiAccess databases, covering the period from the drug’s market introduction to the third quarter of 2024. We used statistical methods including Reporting Odds Ratio (ROR), Proportional Reporting Ratio (PRR), Bayesian Confidence Propagation Neural Network (BCPNN), and Empirical Bayesian Geometric Mean (EBGM) to analyze the association between azacitidine and documented AEs.

**Results:**

The investigation unveiled 16,056 azacitidine-related adverse event (AE) reports from FAERS and 19,867 reports from WHO-VigiAccess. The median duration for the occurrence of these AEs during the observation period was 36 days, with an interquartile range (IQR) spanning from 11 to 126 days. Our statistical analysis identified 27 organ systems associated with AEs induced by azacitidine. Among these, the notable System Organ Classes (SOCs) that met four specific criteria included: infections and infestations, blood and lymphatic system disorders, and neoplasms benign, malignant, and unspecified (including cysts and polyps). Four algorithms identified 443 significant disproportionality preferred terms (PTs), including previously unreported AEs such as death, sepsis, septic shock, respiratory failure, cardiac failure, tumor lysis syndrome, bone marrow failure, interstitial lung disease, and pericarditis. Analysis from the WHO-VigiAccess database showed a ROR of 3.65 and a PRR of 3.30 for the SOC of infections and infestations.

**Conclusion:**

This research not only confirms the widely acknowledged AEs linked to azacitidine but also uncovers several potentially new safety concerns noted in actual clinical practice. These results may offer important vigilance information for clinicians and pharmacists when addressing safety issues associated with azacitidine.

## Introduction

Myelodysplastic syndrome (MDS) and acute myeloid leukemia (AML) are hematologic malignancies that are extremely challenging to manage in the clinic. MDS constitute a group of clonal, heterogeneous bone marrow diseases characterized by disturbed myeloid differentiation and a propensity for clonal evolution and AML transformation. Recurrent mutations in genes encoding, e.g., epigenetic regulation, splicing and signaling are essential in the pathogenesis of MDS and may contribute to the aberrant expression profiles described in the disease (Bersanelli et al., 2021). DNA methylation is crucial for normal biological processes such as imprinting and X inactivation. In MDS and AML, abnormal DNA methylation, especially hypermethylation of promoter regions of certain genes, is prevalent. This leads to the silencing of tumor suppressor genes, contributing to the malignant transformation and progression of the diseases (Gros et al., 2012). For instance, the silencing of key genes due to DNA hypermethylation disrupts normal hematopoiesis and promotes the development of MDS and its progression to AML.

Azacitidine is a cytosine analog and an inhibitor of DNA methyl transferase (DNMT). It is incorporated into newly synthesized DNA after being converted by ribonucleotide reductase. Once incorporated, it inactivates DNMTs, resulting in DNA hypomethylation. In high-risk MDS patients, treatment with azacitidine has been shown to reduce the methylation of the phosphoinositide-phospholipase C β1 (PI-PLCβ1) promoter and reactivate the expression of PI-PLCβ1 mRNA (Sekeres and Taylor, 2022; Kuendgen et al., 2018). This hypomethylation effect can reverse the epigenetic changes associated with MDS and AML, potentially restoring normal gene expression patterns.

Azacitidine also exhibits cytotoxic effects on abnormal hematopoietic cells in the bone marrow. It can be incorporated into RNA by uridine-cytidine kinase, disrupting mRNA and protein synthesis. Additionally, its cytotoxic mechanisms may involve induction of apoptosis and activation of DNA damage pathways. *In vitro* studies indicate that it mainly affects rapidly dividing cells, while non-proliferating cells are relatively insensitive. However, the relative contribution of these cytotoxic effects compared to DNA hypomethylation in determining clinical outcomes remains to be further elucidated.

Azacitidine is approved in the EU for use in patients with higher-risk MDS and acute AML and is approved for all types of MDS in the US. Elderly patients with MDS, who often cannot tolerate intensive chemotherapy, are the main beneficiaries of this treatment. In clinical trials, azacitidine has been demonstrated to reduce the risk of progression to AML in MDS patients compared to conventional care regimens. This is a significant advantage as the progression to AML is a major concern in MDS management. Azacitidine can reduce the need for blood transfusions in MDS patients by improving hematopoiesis and reducing the severity of cytopenias. This helps to improve the quality of life of patients. Even in patients who do not achieve a complete remission (CR), it can still improve survival by stabilizing the disease and improving hematopoiesis (Issa and Kantarjian, 2005).

The Food and Drug Administration (FDA) Adverse Event Reporting System (FAERS) database, known as the largest open drug vigilance database globally, provides comprehensive details on all medications marketed in the United States, in addition to extensive demographic information about users. In contrast to the adverse reaction (AE) literature available in other databases like PubMed, EMBASE, and MEDLINE, AEs within the FAERS database are documented and analyzed individually, making the data more foundational. This database is continuously updated and can be accessed publicly through the official FDA website, which aids in the detection of emerging AE signals. Many research studies have utilized this database to examine AEs linked to clinical usage of drugs (Shu et al., 2022; Guan et al., 2022). Meanwhile, WHO-VigiAccess, a global pharmacovigilance database maintained by the World Health Organization, aggregates AE reports from myriad countries and regions, providing a global perspective on drug safety information (Sultana et al., 2020). Earlier clinical trials and guidelines concerning azacitidine have highlighted the most frequently reported AEs related to its use, including respiratory tract infection, pyrexia, nausea, vomiting, diarrhea, constipation, pneumonia, anaemia, thrombocytopenia, leukopenia, neutropenia, febrile neutropenia, injection site reaction, rigors, weakness, petechiae and hypokalemia.

Nevertheless, the safety profile of azacitidine in real-world, large sample populations, especially regarding the timing of onset for AEs related to its administration, remains uncertain. This research intends to perform a thorough analysis of the FAERS and WHO-VigiAccess databases to investigate AEs associated with azacitidine and to identify possible safety signals in actual clinical settings, thus laying the groundwork for safe medication practices. It is crucial to acknowledge that a considerable portion of the original AE data is reported by patients themselves, which may introduce bias into the study findings.

## Materials and methods

### Data sources, management, and study design

The current investigation employed data sourced from the publicly available FAERS and WHO-VigiAccess databases. FAERS database relies on voluntary report submissions, predominantly from consumers, pharmacists, and healthcare providers (Fang et al., 2023). The study concentrated on all adverse event reports that identified azacitidine as the primary suspected medication, covering the timeframe from the first quarter of 2004 to the third quarter of 2024. During the data management phase, duplicate entries were eliminated, and the terminology associated with adverse events was standardized. The protocol for managing duplicates followed the guidelines set forth by the FDA. In particular, for reports sharing the same case identifiers (CASEIDs), only those with the latest FDA receipt date (FDA_DT) were kept. In instances where both CASEID and FDA_DT were identical, the report with the highest PRIMARYID (the unique identifier for each report) was chosen. Furthermore, WHO-VigiAccess data were gathered from https://www.vigiaccess.org and included a variety of age groups, genders, reporting years, and geographic regions. Both databases used MedDRA (version 26.1) to standardize terminology, ensuring consistency in analysis (Brown, 2004; Vogel et al., 2020).

### Statistical analysis

An extensive examination was performed to highlight the features of adverse event reports associated with azacitidine. In our investigation, we employed both frequentist methods [reporting odds ratio (ROR) (de Leeuw et al., 2002) and proportional reporting ratio (PRR) (Evans et al., 2001)] as well as Bayesian strategies [information component (IC) (Bate et al., 1998) and empirical Bayes geometric mean (EBGM) (Szarfman et al., 2002)] for disproportionality assessment aimed at identifying potential adverse event signals connected to azacitidine. This approach was intended to validate our findings and reduce the occurrence of false-positive safety notifications. The detailed two-by-two contingency tables are presented in Table 1. Moreover, the specific equations and criteria relevant to the four algorithms are depicted in Table 2. In our study, signals indicative of drug-related adverse events were recognized by incorporating those with at least three adverse event records associated with the drugs of interest, and only those signals that fulfilled all four aforementioned algorithm criteria were deemed significant positive indicators (Shu et al., 2023). The entire data processing and statistical evaluation were executed using SAS 9.4 (SAS Institute Inc., Cary, NC, United States), Microsoft EXCEL Professional Plus 2013, and GraphPad Prism 8.0 (GraphPad Software, CA, United States).

**TABLE 1:** Two-by-two contingency table for disproportionality analyses.

Item	Target AEs	Non-target AEs	Total
Target drug	a	b	a+b
Other drugs	c	d	c + d
Total	a+c	b + d	a+b + c + d

**TABLE 2:** Four primary algorithms used for signal detection.

Methods	Formula	Signal standard
ROR	$\text{ROR}=\frac{a\;/\;c}{b\;/\;d}$ $SE\left(lnROR\right)=\sqrt{\frac{1}{a}+\frac{1}{b}+\frac{1}{c}+\frac{1}{d}}$ $95\%CI=e^{\ln\left(ROR\right)\pm 1.96se}$	95% CI > 1, a≥3
PRR	$SE\left(lnPRR\right)=\sqrt{\frac{1}{a}-\frac{1}{a+b}+\frac{1}{c}-\frac{1}{c+d}}$	PRR≥2, χ^2^ ≥ 4, a≥3
BCPNN	$95\%CI=e^{\ln\left(PRR\right)\pm 1.96se}$	IC025 > 0
MGPS	$\text{EBGM}=\frac{a\left(a+b+c+d\right)}{\left(a+c\right)\left(a+b\right)}$ $SE\left(lnEBGM\right)=\sqrt{\frac{1}{a}+\frac{1}{b}+\frac{1}{c}+\frac{1}{d}}$ $95\%CI=e^{\ln\left(EBGM\right)\pm 1.96se}$	EBGM05 > 2

Abbreviation: ROR, reporting odds ratio; PRR, proportional reporting ratio; BCPNN, bayesian confidence propagation neural network; MGPS, multi-item gamma Poisson shrinker; EBGM, empirical Bayesian geometric mean; CI, confidence interval; χ^2^, chi-squared; IC, information component; IC025, the lower limit of the 95% one-sided CI, of the IC; EBGM05, the lower 95% onesided CI, of EBGM.

## Results

### Descriptive analysis

**FIGURE 1:**
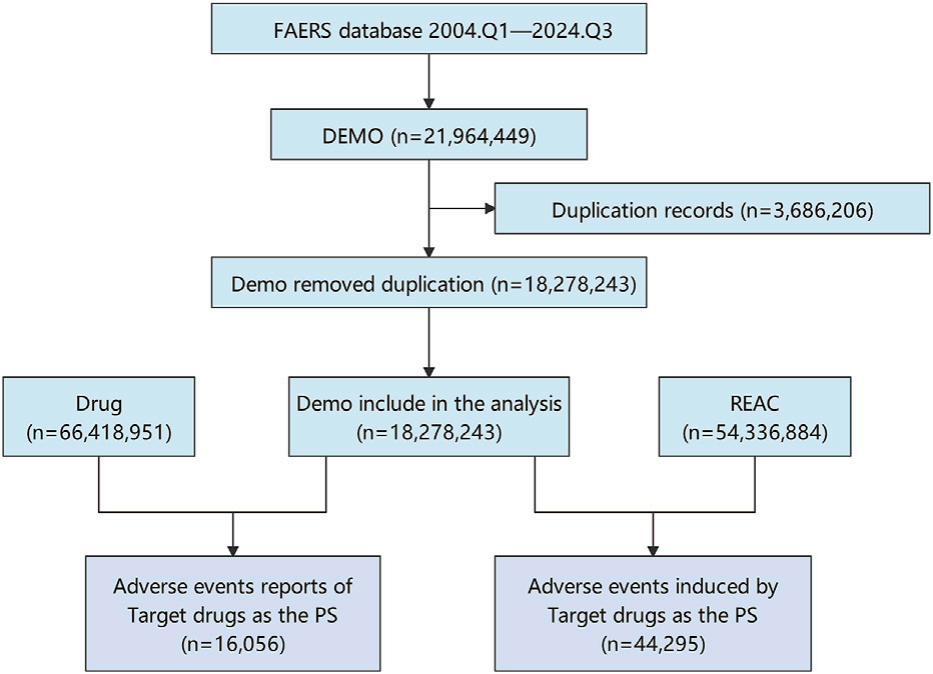
Flow diagram for the selection of AEs associated with azacitidine from FAERS database.

**FIGURE 2:**
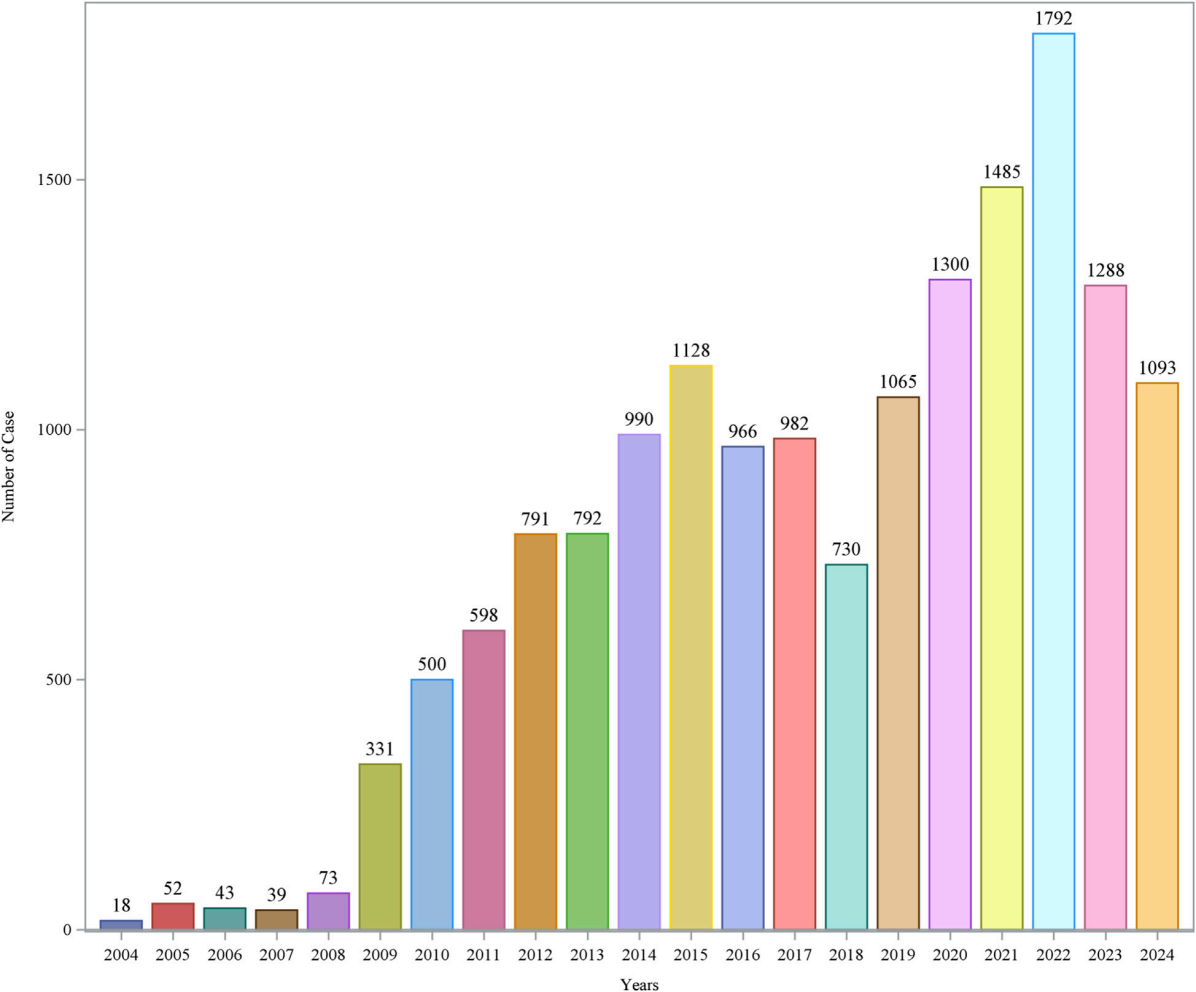
Reports number and trends of azacitidine-related AEs.

The comprehensive FAERS dataset, which spans from Q1 2004 to Q3 2024, contains a total of 21,964,449 entries. After the removal of duplicates, 16,056 reports associated with azacitidine were analyzed, encompassing 44,295 adverse events. Details regarding the data collection, interpretation, and analysis processes are illustrated in Figure 1. The clinical attributes of events related to azacitidine are summarized in Table 3. Demographic data indicate that 53.53% of the adverse events occurred in males, while 32.53% were associated with female patients. The predominant age group consisted of individuals aged 65 and older, accounting for 55.36% of the overall cases. In terms of reporting sources, health professionals, including physicians (37.69%), pharmacists (24.39%), and other health professionals (27.01%), submitted 89.09% of the adverse event reports. Among the countries reporting adverse events, the United States contributed the highest number of reports (n = 4,239, 26.40%), followed by Japan (n = 1,971, 12.28%) and France (n = 1,230, 7.66%). Furthermore, a significant proportion of patients (n = 15,379, 95.78%) experienced serious outcomes, which included other serious medical events (n = 7,280, 45.34%), hospitalization (n = 7,075, 44.06%), and death (n = 6,562, 40.87%). As depicted in Figure 2, the year 2022 recorded the highest number of reports (n = 1,792), with subsequent years exhibiting varying frequencies. According to the data from the WHO-VigiAccess database, the earliest recorded adverse reaction to azacitidine dates back to 1978. By 2024, the WHO had accumulated a cumulative total of 19,867 reports related to ADRs for azacitidine. Further details regarding the analysis of these reports are presented in Supplementary Table S1.

**TABLE 3:** An overview of essential demographic and clinical details regarding reports linked to azacitidine, derived from the FAERS database (From the first quarter of 2004 to the third quarter of 2024).

Characteristics	Number of cases	Caseproportion,%
Number of events	16,056	
Sex		
Female	5,223	32.53
Male	8,594	53.53
Not Specified	2,239	13.94
Age		
<18 (%)	263	1.64
18–44 (%)	510	3.18
45–64 (%)	2,375	14.79
≥65 (%)	8,889	55.36
NotSpecified (%)	4,019	25.03
Reporter		
Consumer	1,289	8.03
Physician	6,051	37.69
Pharmacist	3,916	24.39
Not Specified	461	2.87
Lawyer	2	0.01
Other health-professional	4,337	27.01
Top 5 reporting countries		
United States	4,239	26.40
Japan	1,971	12.28
France	1,230	7.66
Spain	1,058	6.59
Germany	972	6.05
AE Severity		
Serious	15,379	95.78
Non-Serious	677	4.22
Serious Outcome		
Life-Threatening	1,741	10.84
Hospitalization	7,075	44.06
Disability	289	1.80
Death	6,562	40.87
Congenital Anomaly	5	0.03
Required Intervention to Prevent Permanent Impairment/Damage	29	0.18
Other Serious Medical Events	7,280	45.34

### Distribution of adverse events at the system organ class (SOC) level

**FIGURE 3:**
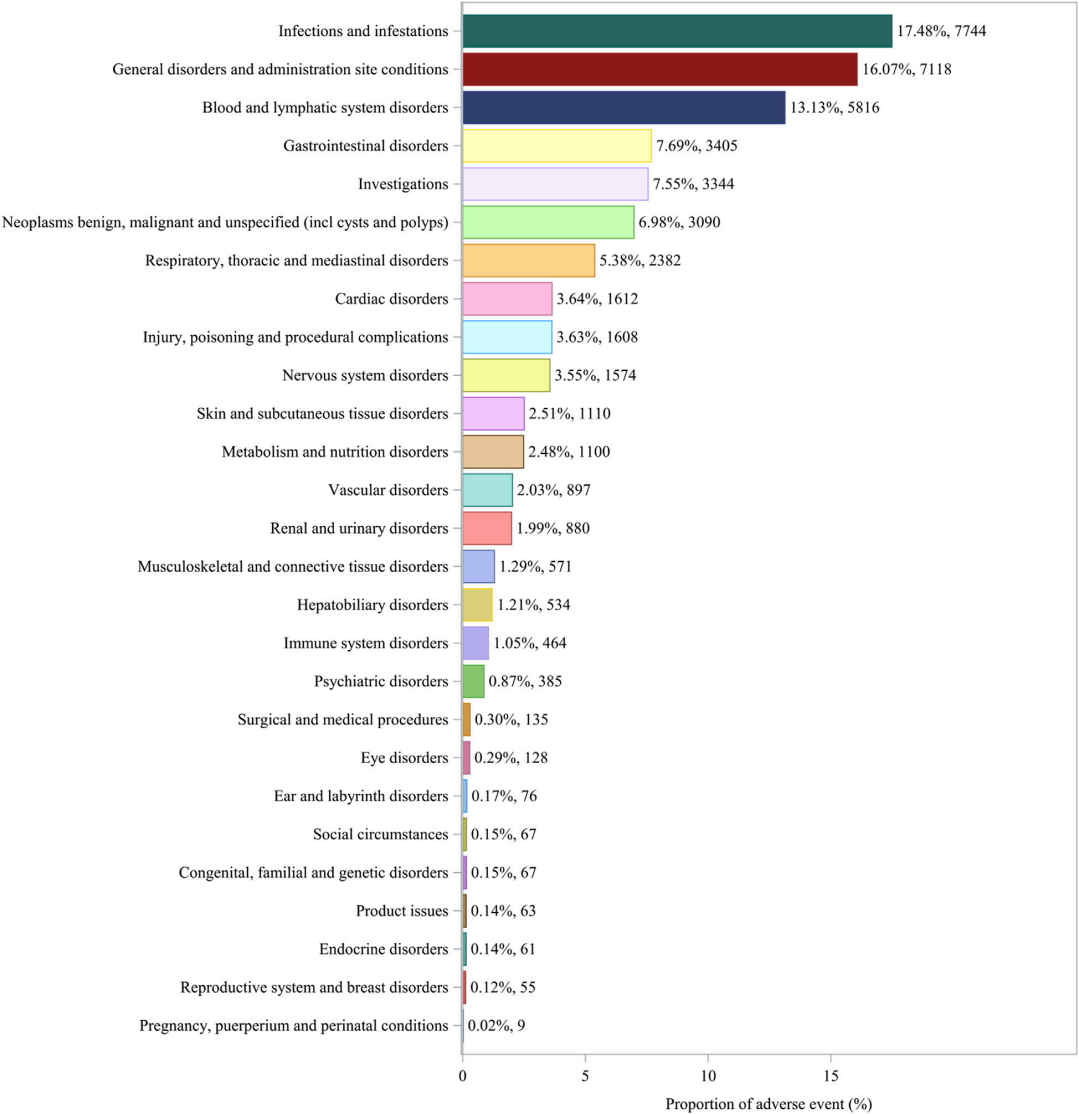
Proportion of adverse events by system organ class for azacitidine.

The percentage of affirmative signals for AEs associated with azacitidine at the SOC level is illustrated in Figure 3. Additionally, the specific strength of signals for azacitidine at the SOC level is elaborated in Table 4. We statistically identified 27 organ systems linked to AEs induced by azacitidine. Notably, the important SOCs that satisfied four specified criteria included infections and infestations (SOC: 10021881, n = 7,744), blood and lymphatic system disorders (SOC: 10005329, n = 5,816), and neoplasms benign, malignant and unspecified (incl cysts and polyps) (SOC: 10029104, n = 3,090). Blood and lymphatic system disorders presented the highest signal, while infections and infestations were the most frequently reported SOC. For the SOC of infections and infestations, the WHO-VigiAccess database revealed a ROR of 3.65 (95% CI: 3.55–3.75) and a PRR of 3.30 (95% CI: 3.22–3.38), with detailed results presented in Supplementary Table S2.

**TABLE 4:** Signal strength of azacitidine-related adverse events across SOCs in the FDA Adverse Event Reporting System database.

SOC	Case number	ROR (95% CI)	PRR (95% CI)	chi_square	IC (IC025)	EBGM (EBGM05)
Infections and infestations*	7744	3.84 (3.75, 3.94)	3.35 (3.28, 3.42)	13409.5	1.74 (1.70)	3.34 (3.26)
General disorders and administration site conditions	7118	0.91 (0.88, 0.93)	0.92 (0.90, 0.94)	57.69	−0.12 (−0.15)	0.92 (0.90)
Blood and lymphatic system disorders*	5816	8.87 (8.62, 9.11)	7.83 (7.65, 8.02)	35036.7	2.96 (2.92)	7.79 (7.58)
Gastrointestinal disorders	3405	0.90 (0.86, 0.93)	0.90 (0.87, 0.93)	38.26	−0.15 (−0.20)	0.90 (0.87)
Investigations	3344	1.25 (1.20, 1.29)	1.23 (1.19, 1.27)	150.63	0.30 (0.24)	1.23 (1.19)
Neoplasms benign, malignant and unspecified (incl cysts and polyps) *	3090	2.77 (2.67, 2.88)	2.65 (2.56, 2.74)	3252.57	1.40 (1.35)	2.65 (2.55)
Respiratory, thoracic and mediastinal disorders	2382	1.15 (1.10, 1.20)	1.14 (1.10, 1.19)	44.77	0.19 (0.13)	1.14 (1.10)
Cardiac disorders	1612	1.39 (1.33, 1.46)	1.38 (1.31, 1.45)	171.87	0.46 (0.39)	1.38 (1.31)
Injury, poisoning and procedural complications	1608	0.33 (0.31, 0.34)	0.35 (0.33, 0.37)	2148.96	−1.51 (−1.58)	0.35 (0.33)
Nervous system disorders	1574	0.40 (0.38, 0.42)	0.42 (0.40, 0.44)	1397.37	−1.26 (−1.33)	0.42 (0.40)
Skin and subcutaneous tissue disorders	1110	0.45 (0.43, 0.48)	0.47 (0.44, 0.49)	718.75	−1.10 (−1.19)	0.47 (0.44)
Metabolism and nutrition disorders	1100	1.15 (1.08, 1.22)	1.14 (1.08, 1.21)	20.41	0.19 (0.11)	1.14 (1.08)
Vascular disorders	897	0.95 (0.89, 1.01)	0.95 (0.89, 1.01)	2.63	−0.08 (−0.17)	0.95 (0.89)
Renal and urinary disorders	880	1.04 (0.97, 1.11)	1.04 (0.98, 1.11)	1.45	0.06 (−0.04)	1.04 (0.97)
Musculoskeletal and connective tissue disorders	571	0.24 (0.22, 0.26)	0.25 (0.23, 0.27)	1365.92	−2.01 (−2.13)	0.25 (0.23)
Hepatobiliary disorders	534	1.32 (1.21, 1.44)	1.31 (1.21, 1.43)	40.54	0.39 (0.27)	1.31 (1.21)
Immune system disorders	464	0.95 (0.87, 1.04)	0.95 (0.87, 1.04)	1.08	−0.07 (−0.20)	0.95 (0.87)
Psychiatric disorders	385	0.15 (0.13, 0.16)	0.15 (0.14, 0.17)	1902.21	−2.70 (−2.85)	0.15 (0.14)
Surgical and medical procedures	135	0.22 (0.19, 0.27)	0.23 (0.19, 0.27)	360.76	−2.14 (−2.38)	0.23 (0.19)
Eye disorders	128	0.14 (0.12, 0.17)	0.15 (0.12, 0.17)	657.82	−2.78 (−3.03)	0.15 (0.12)
Ear and labyrinth disorders	76	0.39 (0.31, 0.49)	0.39 (0.32, 0.49)	70.86	−1.34 (−1.66)	0.39 (0.32)
Congenital, familial and genetic disorders	67	0.50 (0.39, 0.63)	0.50 (0.39, 0.64)	33.53	−1.00 (−1.34)	0.50 (0.39)
Social circumstances	67	0.32 (0.26, 0.41)	0.33 (0.26, 0.41)	94.11	−1.62 (−1.95)	0.33 (0.26)
Product issues	63	0.09 (0.07, 0.11)	0.09 (0.07, 0.11)	604.00	−3.50 (−3.84)	0.09 (0.07)
Endocrine disorders	61	0.54 (0.42, 0.70)	0.54 (0.42, 0.70)	23.57	−0.88 (−1.24)	0.54 (0.42)
Reproductive system and breast disorders	55	0.14 (0.11, 0.18)	0.14 (0.11, 0.18)	295.89	−2.85 (−3.21)	0.14 (0.11)
Pregnancy, puerperium and perinatal conditions	9	0.05 (0.02, 0.09)	0.05 (0.02, 0.09)	174.13	−4.41 (−5.17)	0.05 (0.02)

Asterisks (*) indicate significant signals in four algorithms. PRR, proportional reporting ratio; ROR, reported odds ratio; IC, information component; EBGM, the empirical Bayes geometric mean; IC025 and EBGM05, lower limit of the 95% two-sided confidence interval for IC and EBGM, respectively. Signals are detected when all the following criteria are met: PRR ≥ 2 and χ^2^ > 4, lower limit of 95% CI of ROR >1, IC025 > 0, EBGM05 > 2.

### Distribution of adverse events at the preferred term (PT) level

Supplementary Table S3 showed 443 PTs that met all four algorithm criteria at the PT level. In the table we can find respiratory tract infection (PT: 10062352), pyrexia (PT: 10037660),pneumonia (PT: 10035664), anaemia (PT: 10002034), thrombocytopenia (PT: 10043554), platelet count decreased (PT: 10035528), leukopenia (PT: 10024384), neutropenia (PT: 10029354), febrile neutropenia (PT: 10016288), and injection site reaction (PT: 10022095), which were consistent with the label for azacitidine.

These 443 PTs were ranked according to report number and the top 30 PTs in terms of number of reports were selected for inclusion in Table 5, which showed that the top 5 PTs in terms of number of morbidities were death (n = 1,943), febrile neutropenia (n = 1,622), pneumonia (n = 1432), acute myeloid leukaemia (n = 1,117), and Neutropenia (n = 843). Moreover, we ranked the PTs in Supplementary Table S3 according to the strength of the EBGM algorithm, and finally obtained the top 30 PTs in terms of signal strength to be included in Table 6. The results showed that the top 5 in terms of signal strength were angioimmunoblastic T-cell lymphoma refracto (n = 3, EBGM = 525.73), FLT3 gene mutation (n = 5, EBGM = 438.11), myelodysplastic syndrome transformation (n = 120, EBGM = 275.66), juvenile chronic myelomonocytic leukaemia (n = 9, EBGM = 234.90), transformation to acute myeloid leukaemia (n = 91, EBGM = 213.03).

**TABLE 5:** The top 30 PTs of azacitidine selected based on a level of 443 PTs that met the four algorithmic criteria.

SOC	PT	Case number	ROR (95% CI)	PRR (95% CI)	chi_square	IC (IC025)	EBGM (EBGM05)
General disorders and administration site conditions	Death	1943	3.28 (3.13, 3.43)	3.18 (3.04, 3.32)	2932.817023	1.67 (1.60)	1.66 (1.59)
Blood and lymphatic system disorders	Febrile neutropenia	1622	37.36 (35.53, 39.29)	36.03 (34.32, 37.82)	53717.17939	5.13 (5.03)	5.12 (5.03)
Infections and infestations	Pneumonia	1432	6.07 (5.76, 6.40)	5.91 (5.61, 6.22)	5841.830724	2.56 (2.47)	2.55 (2.47)
Neoplasms benign, malignant and unspecified (incl cysts and polyps)	Acute myeloid leukaemia	1117	112.47 (105.72, 119.66)	109.66 (103.22, 116.50)	110418.3137	6.65 (6.44)	6.59 (6.49)
Blood and lymphatic system disorders	Neutropenia	843	9.08 (8.48, 9.72)	8.93 (8.35, 9.55)	5902.737155	3.15 (3.03)	3.14 (3.03)
Infections and infestations	Sepsis	838	10.65 (9.95, 11.41)	10.47 (9.79, 11.20)	7131.501172	3.38 (3.26)	3.37 (3.26)
General disorders and administration site conditions	Pyrexia	819	3.33 (3.10, 3.56)	3.28 (3.07, 3.51)	1304.50997	1.71 (1.61)	1.71 (1.60)
Blood and lymphatic system disorders	Thrombocytopenia	768	9.91 (9.22, 10.64)	9.75 (9.09, 10.46)	5995.410102	3.28 (3.15)	3.27 (3.15)
Blood and lymphatic system disorders	Anaemia	685	4.99 (4.63, 5.38)	4.93 (4.57, 5.31)	2142.611524	2.30 (2.18)	2.29 (2.17)
Infections and infestations	Infection	620	6.31 (5.83, 6.83)	6.23 (5.76, 6.74)	2716.290874	2.63 (2.50)	2.63 (2.50)
Investigations	Platelet count decreased	513	6.77 (6.20, 7.38)	6.70 (6.15, 7.30)	2478.118124	2.74 (2.59)	2.73 (2.58)
Neoplasms benign, malignant and unspecified (incl cysts and polyps)	Myelodysplastic syndrome	502	49.48 (45.24, 54.12)	48.93 (44.78, 53.47)	22670.1493	5.56 (5.30)	5.49 (5.35)
Blood and lymphatic system disorders	Pancytopenia	456	11.72 (10.68, 12.86)	11.61 (10.59, 12.73)	4384.768445	3.53 (3.36)	3.51 (3.35)
Infections and infestations	Septic shock	415	14.01 (12.71, 15.44)	13.89 (12.62, 15.29)	4912.122783	3.78 (3.59)	3.76 (3.60)
Investigations	White blood cell count decreased	395	5.08 (4.60, 5.61)	5.04 (4.57, 5.57)	1277.985885	2.33 (2.17)	2.32 (2.16)
General disorders and administration site conditions	Disease progression	354	4.28 (3.85, 4.75)	4.25 (3.83, 4.71)	878.1746501	2.08 (1.92)	2.08 (1.90)
Investigations	Neutrophil count decreased	324	11.70 (10.48, 13.05)	11.62 (10.42, 12.96)	3116.91617	3.53 (3.32)	3.50 (3.32)
General disorders and administration site conditions	Therapy non-responder	291	7.60 (6.77, 8.53)	7.55 (6.73, 8.47)	1645.695229	2.91 (2.71)	2.89 (2.70)
Respiratory, thoracic and mediastinal disorders	Respiratory failure	247	4.68 (4.13, 5.30)	4.66 (4.11, 5.27)	707.4061117	2.22 (2.01)	2.20 (1.99)
General disorders and administration site conditions	General physical health deterioration	245	3.17 (2.80, 3.60)	3.16 (2.79, 3.58)	361.968464	1.66 (1.46)	1.65 (1.44)
Blood and lymphatic system disorders	Cytopenia	239	33.03 (29.04, 37.57)	32.86 (28.91, 37.35)	7191.177544	5.00 (4.64)	4.91 (4.70)
Investigations	Haemoglobin decreased	215	2.85 (2.50, 3.26)	2.84 (2.49, 3.25)	257.0028506	1.51 (1.30)	1.50 (1.27)
Neoplasms benign, malignant and unspecified (incl cysts and polyps)	Acute myeloid leukaemia recurrent	201	170.44 (147.02, 197.59)	169.67 (146.45, 196.58)	29606.29149	7.22 (6.21)	6.77 (6.54)
Cardiac disorders	Cardiac failure	190	3.29 (2.85, 3.79)	3.28 (2.84, 3.78)	300.3460119	1.71 (1.48)	1.70 (1.46)
Respiratory, thoracic and mediastinal disorders	Pneumonitis	180	9.81 (8.47, 11.37)	9.78 (8.45, 11.32)	1407.933343	3.28 (3.00)	3.24 (3.00)
General disorders and administration site conditions	Multiple organ dysfunction syndrome	172	5.36 (4.62, 6.23)	5.35 (4.60, 6.21)	605.7185542	2.41 (2.16)	2.40 (2.14)
Respiratory, thoracic and mediastinal disorders	Pleural effusion	164	3.68 (3.16, 4.30)	3.67 (3.15, 4.28)	318.5215644	1.87 (1.63)	1.86 (1.60)
Blood and lymphatic system disorders	Myelosuppression	161	9.33 (7.99, 10.90)	9.30 (7.97, 10.86)	1184.109951	3.21 (2.91)	3.17 (2.91)
Infections and infestations	Cellulitis	158	4.28 (3.66, 5.01)	4.27 (3.65, 4.99)	394.5485802	2.09 (1.83)	2.08 (1.81)
Nervous system disorders	Cerebral haemorrhage	158	6.09 (5.21, 7.12)	6.07 (5.19, 7.10)	666.3500187	2.60 (2.32)	2.57 (2.31)

**TABLE 6:** Top 30 azacitidine PTs out of 260 meeting four algorithmic criteria, ranked by EBGM.

SOC	PT	Case number	ROR (95%CI)	PRR (95%CI)	chi_square	IC (IC025)	EBGM (EBGM05)
Neoplasms benign, malignant and unspecified (incl cysts and polyps)	Angioimmunoblastic T-cell lymphoma refractory	3	919.34 (205.75–4,107.87)	919.28 (205.75, 4107.36)	1572.479519	9.04 (0.22)	525.73 (117.66)
Congenital, familial and genetic disorders	FLT3 gene mutation	5	681.02 (228.22–2032.20)	680.95 (228.22, 2031.81)	2,182.335076	8.78 (1.17)	438.11 (146.82)
Neoplasms benign, malignant and unspecified (incl cysts and polyps)	Myelodysplastic syndrome transformation	120	356.24 (290.67–436.60)	355.28 (290.01, 435.24)	32867.22805	8.11 (6.11)	275.66 (224.92)
Neoplasms benign, malignant and unspecified (incl cysts and polyps)	Juvenile chronic myelomonocytic leukaemia	9	290.36 (140.39–600.50)	290.30 (140.38, 600.31)	2097.858358	7.88 (2.26)	234.90 (113.58)
Neoplasms benign, malignant and unspecified (incl cysts and polyps)	Transformation to acute myeloid leukaemia	91	258.12 (205.87–323.65)	257.60 (205.52, 322.86)	19220.42859	7.73 (5.68)	213.03 (169.90)
Infections and infestations	Pseudomonal skin infection	3	229.84 (66.97–788.82)	229.82 (66.97,788.71)	575.5555181	7.60 (0.40)	193.69 (56.43)
Gastrointestinal disorders	Ulcerative duodenitis	4	204.30 (70.88–588.85)	204.28 (70.88, 588.74)	693.5626747	7.45 (0.89)	175.24 (60.80)
Neoplasms benign, malignant and unspecified (incl cysts and polyps)	Blastic plasmacytoid dendritic cell neoplasia	9	183.89 (91.26–370.57)	183.86 (91.25, 370.45)	1423.262916	7.32 (2.27)	160.00 (79.40)
Hepatobiliary disorders	Portal vein cavernous transformation	5	180.27 (70.50–460.95)	180.25 (70.50, 460.85)	777.0149229	7.30 (1.28)	157.27 (61.51)
Neoplasms benign, malignant and unspecified (incl cysts and polyps)	Acute myeloid leukaemia refractory	16	173.61 (102.84–293.08)	173.55 (102.82, 292.93)	2404.461653	7.25 (3.20)	152.15 (90.13)
Neoplasms benign, malignant and unspecified (incl cysts and polyps)	Acute myeloid leukaemia recurrent	201	170.44 (147.02–197.59)	169.67 (146.45, 196.58)	29606.29149	7.22 (6.21)	149.16 (128.67)
Skin and subcutaneous tissue disorders	Neutrophilic panniculitis	8	166.23 (79.43–347.88)	166.20 (79.42, 347.78)	1156.776647	7.19 (2.07)	146.47 (69.99)
Investigations	Blast cell count increased	96	147.03 (118.96–181.73)	146.72 (118.75, 181.27)	12408.48473	7.03 (5.50)	131.14 (106.10)
Nervous system disorders	Lower motor neurone lesion	5	145.93 (57.73–368.87)	145.92 (57.73, 368.79)	643.0655836	7.03 (1.28)	130.50 (51.63)
Infections and infestations	Malassezia infection	4	132.52 (47.23–371.81)	132.51 (47.23, 371.75)	471.1318643	6.90 (0.91)	119.68 (42.66)
Neoplasms benign, malignant and unspecified (incl cysts and polyps)	Chronic myelomonocytic leukaemia	58	131.09 (99.98–171.88)	130.92 (99.88, 171.61)	6756.352788	6.89 (4.91)	118.38 (90.29)
Social circumstances	Blood product transfusion dependent	34	114.26 (80.39–162.40)	114.18 (80.35, 162.24)	3489.25697	6.71 (4.21)	104.53 (73.55)
Neoplasms benign, malignant and unspecified (incl cysts and polyps)	Acute erythroid leukaemia	10	113.52 (59.38–217.00)	113.49 (59.38, 216.92)	1020.51056	6.70 (2.42)	103.96 (54.38)
Neoplasms benign, malignant and unspecified (incl cysts and polyps)	Acute myeloid leukaemia	1117	112.47 (105.72–119.66)	109.66 (103.22, 116.50)	110418.3137	6.65 (6.44)	100.74 (94.69)
Neoplasms benign, malignant and unspecified (incl cysts and polyps)	Leukaemic infiltration pulmonary	3	102.15 (31.46–331.72)	102.14 (31.46, 331.67)	277.3437763	6.56 (0.44)	94.36 (29.06)
Infections and infestations	Protothecosis	5	100.48 (40.37–250.07)	100.47 (40.37, 250.02)	455.086203	6.54 (1.28)	92.93 (37.34)
Infections and infestations	Gastroenteritis astroviral	4	100.07 (36.11–277.30)	100.06 (36.11, 277.25)	362.6651429	6.53 (0.91)	92.58 (33.41)
General disorders and administration site conditions	Injection site vasculitis	4	96.14 (34.75–266.02)	96.13 (34.75, 265.98)	349.1893898	6.48 (0.91)	89.21 (32.24)
Infections and infestations	Emphysematous cholecystitis	3	94.29 (29.14–305.13)	94.28 (29.14, 305.09)	257.1093197	6.45 (0.44)	87.62 (27.08)
General disorders and administration site conditions	Administration site induration	5	91.48 (36.87–226.97)	91.47 (36.87, 226.93)	416.3377669	6.41 (1.28)	85.19 (34.33)
Vascular disorders	Venous aneurysm	3	89.69 (27.77–289.66)	89.69 (27.77, 289.61)	245.1527807	6.39 (0.44)	83.64 (25.90)
Infections and infestations	Sphingomonas paucimobilis infection	3	89.69 (27.77–289.66)	89.69 (27.77, 289.61)	245.1527807	6.39 (0.44)	83.64 (25.90)
Neoplasms benign, malignant and unspecified (incl cysts and polyps)	Leukaemia cutis	8	85.28 (41.65–174.63)	85.27 (41.64, 174.58)	622.8948207	6.32 (2.03)	79.79 (38.96)
Surgical and medical procedures	Allogenic stem cell transplantation	7	78.73 (36.66–169.06)	78.71 (36.66, 169.02)	504.6840321	6.21 (1.81)	74.03 (34.47)
Infections and infestations	Disseminated trichosporonosis	3	78.24 (24.35–251.39)	78.24 (24.35, 251.35)	215.0230856	6.20 (0.44)	73.60 (22.91)

In addition to the common AEs explicitly mentioned with the specification, we also identified suspected AEs not mentioned in the specification, such as death (n = 1,943), acute myeloid leukaemia (n = 1,117), sepsis (n = 838), infection (n = 620), myelodysplastic syndrome (n = 502), septic shock (n = 415), Respiratory failure (n = 247), cardiac failure (n = 190), tumour lysis syndrome (n = 137), bone marrow failure (n = 136),interstitial lung disease (n = 124), pericarditis (n = 114). Other unexpected PTs in drug instructions were displayed in Supplementary Table S3.

### Time-to-onset analysis

**FIGURE 4:**
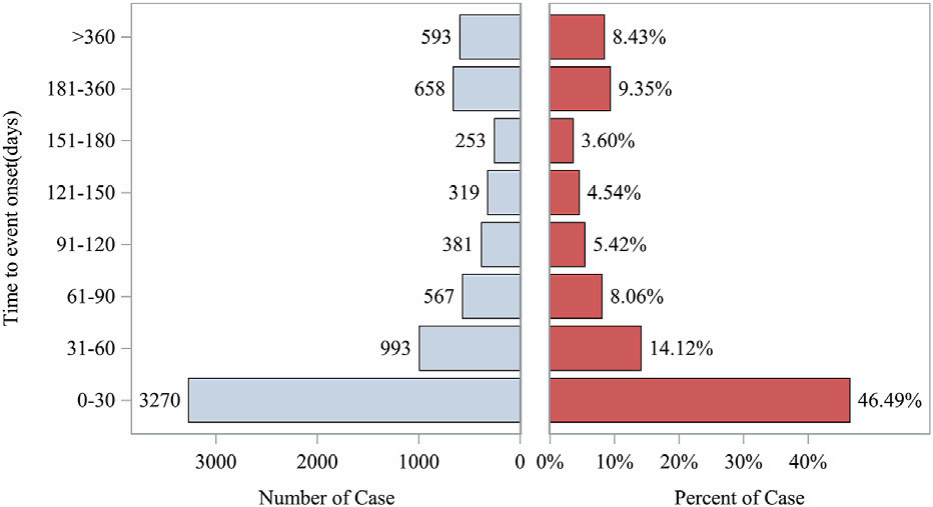
Time to onset of adverse events induced by azacitidine.

**FIGURE 5:**
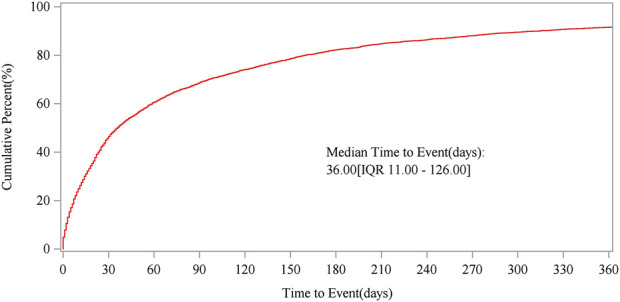
Cumulative incidence of adverse events related to azacitidine over time.

The gathering of onset times for events associated with azacitidine required the removal of reports that had either unreported or incorrect onset times from the analysis. A total of 7,034 cases fulfilled the inclusion requirements, with an average onset time of 130.67 days and a median of 36 days (interquartile range [IQR] 11–126 days). Our data showed that the most onset time of azacitidine-related AEs was less than 30 days (n = 3,270, 46.49%). Of note, AEs might still have occurred after half a year for azacitidine treatment, with a proportion of 17.78%,as depicted in Figure 4. Additionally, Figure 5 demonstrates the cumulative incidence curve for adverse events.

## Discussion

MDS are a group of hematopoietic stem cell malignancies characterized by ineffective hematopoiesis, peripheral cytopenias, and a risk of transforming into AML. Symptoms include infection, bleeding, bruising, and fatigue, with most patients eventually dying from infectious complications or AML transformation. Standard treatments involve supportive care, such as blood transfusions, hematopoietic factors, and antibiotics, while allogeneic stem-cell transplantation is the primary curative option, often unsuitable due to patient age or comorbidities. DNA methylation, an epigenetic mechanism, is crucial for gene silencing without altering the coding sequence. Malignant cells exploit this to silence tumor-suppressor genes (Figueroa et al., 2009). Azacitidine, approved by the FDA in 2004, is the first drug targeting epigenetic gene silencing in MDS, offering a novel therapeutic approach to counteract the malignant phenotype (Mozessohn et al., 2021).

This study represents the first extensive and systematic pharmacovigilance investigation of AEs associated with azacitidine using the FAERS and WHO-VigiAccess databases following its market release. The primary aim of this research is to provide a detailed and comprehensive characterization and analysis of the AEs related to azacitidine reported to date. The results presented in this paper offer valuable and precise insights into the safety profile of azacitidine in a real-world clinical setting.

Our findings regarding the sex ratio of patients indicate that males outnumber females, with proportions of 53.53% and 32.53%, respectively. The predominant age group consists of individuals aged 65 years and older, who account for 55.36% of the overall cases. Bone marrow hyperplasia and abnormal syndromes are most prevalent among individuals over 50 years of age, particularly in those over 65, with a higher incidence observed in men compared to women. This observation aligns with the results of our dataset analysis. Meanwhile, health professionals, including physicians (37.69%), pharmacists (24.39%), and other health professionals (27.01%), submitted 89.09% of the adverse event reports., which might be considered a reliable reporting source. Among the countries reporting AEs, the United States had the highest number of reports, totaling 4,239 (26.40%). This trend may be attributed to a larger population of medication users, as well as factors such as a greater overall population size, a stronger willingness to report, earlier market entry, and an earlier expansion of indications, all of which collectively facilitated the widespread use of the medication. Furthermore, a substantial proportion of patients experienced serious outcomes, with 15,379 cases (95.78%) reporting such events, including other serious medical events (7,280 cases, 45.34%), hospitalization (7,075 cases, 44.06%), and death (6,562 cases, 40.87%). We statistically identified 27 organ systems associated with AEs induced by azacitidine.

The important SOCs that met the four specified criteria included infections and infestations (SOC: 10021881, n = 7,744), blood and lymphatic system disorders (SOC: 10005329, n = 5,816), and neoplasms benign, malignant and unspecified (incl cysts and polyps) (SOC: 10029104, n = 3,090). Blood and lymphatic system disorders exhibited the highest signal, while infections and infestations were the most frequently reported SOC. Among the 443 reported AEs that fulfilled the four established criteria, confirmed cases included respiratory tract infection, pyrexia, pneumonia, anaemia, thrombocytopenia, platelet count decreased, leukopenia, neutropenia, febrile neutropenia, and injection site reaction, all of which were consistent with the label for azacitidine. Furthermore, we identified potential AEs that were not listed on the product’s label, such as death, sepsis, infection, septic shock, respiratory failure, cardiac failure, tumor lysis syndrome, bone marrow failure, and interstitial lung disease, as well as pericarditis. Other unexpected PTs in drug instructions are displayed in Supplementary Table S3.

Furthermore, no significant disproportionate signals were identified for nausea, vomiting, diarrhea, and constipation—adverse effects that are frequently reported in the azacitidine insert. These discrepancies may arise from the fact that AEs are relatively common across all drugs documented in the FAERS database. The substantial volume of AE reports linked to multiple drugs may dilute the signal score. Disproportionality necessitates that drug-specific AEs be reported either with greater or lesser frequency. Consequently, the absence of a signal does not imply that there are no associated AEs; rather, it indicates that these AEs do not appear to be disproportionate (Sakaeda et al., 2013).

For patients at high risk for febrile neutropenia, granulocyte colony-stimulating factor prophylaxis can be administered, as well as for those at moderate risk who present with additional risk factors (Spring et al., 2021). If not managed appropriately, severe hematologic adverse events may lead to complications such as bleeding and potentially secondary infections, which could progress to sepsis. Therefore, clinicians must remain vigilant in the early assessment and management of azacitidine-related hematologic toxicity.

Azacitidine was generally well tolerated in patients with MDS and AML. Most deaths or adverse events leading to drug interruption were attributed to the disease itself or to the consequences of cytopenias, such as sepsis and bleeding. Infectious complications occur more frequently in MDS patients than in non-MDS patients, with infections and related complications being significant contributors to morbidity and mortality in this population (Lee et al., 2011). Among these, pneumonia, sepsis, bacteremia, skin infections, and fungal infections are the most prevalent. Impaired neutrophil function in MDS patients may play a crucial role in their increased susceptibility to infections. In a retrospective study, 59% of 184 patients with high-risk MDS or AML who received azacitidine experienced an infectious event (Radsak et al., 2017). Notably, the incidence of infectious events decreased with an increasing number of azacitidine treatment cycles; however, the risk of infection was higher during the early stages of treatment (Merkel et al., 2013). During the treatment period, it is essential to closely monitor the patient’s blood routine, infection indicators (such as C-reactive protein and blood cultures), and clinical manifestations to promptly detect signs of infection. For high-risk patients, the consideration of prophylactic antibacterial agents, such as oral fluoroquinolones, may be warranted to prevent respiratory infections.

In patients with a history of cardiovascular disease, a cardiac evaluation should be conducted prior to initiating azacitidine, and cardiac function should be monitored periodically throughout the treatment. Azacitidine may play a potential role in the development of heart failure. Research indicates that newly diagnosed AML patients with a history of cardiovascular or pulmonary disease experience a significantly higher rate of cardiac events when treated with azacitidine (Perino et al., 2020). Furthermore, treatment with azacitidine may be linked to the onset of cardiac failure, particularly in patients with a history of cardiac disease and other serious comorbidities. The presence of cardiovascular history and comorbidities influences the severity of cardiac failure, and in some cases, patients may continue to receive azacitidine following adjustments to their cardiac medications.

Azacitidine may exhibit cardiotoxic effects, particularly after ruling out other common causes of pericarditis. Hypomethylating agents can modify gene expression, including genes associated with immune regulation. This alteration may result in abnormal immune system activation, leading to inflammatory responses and immune-mediated cardiac damage, such as pericarditis. At elevated doses, azacitidine is cytotoxic and may directly harm pericardial cells, contributing to the development of pericarditis. Clinicians should remain vigilant regarding the potential risk of pericarditis when prescribing azacitidine. If azacitidine-induced pericarditis is suspected, clinicians should contemplate discontinuing the drug to alleviate the patient’s symptoms and mitigate the risk of developing constrictive pericarditis, pericardial effusion, and cardiac tamponade (Newman et al., 2016).

Azacitidine-induced interstitial pneumonitis represents a potentially serious adverse effect. This medication may lead to drug-induced lung injury in patients with MDS. Notably, interstitial lung disease associated with azacitidine typically resolves with steroid treatment and the discontinuation of the drug (Sekhri et al., 2012; Kuroda et al., 2014). However, further research and confirmation are necessary to elucidate the relationship between azacitidine and interstitial lung disease.

The timing of events related to azacitidine was recorded, with the analysis omitting instances of missing or incorrectly documented onset times. In total, 7,034 cases met the inclusion criteria, indicating a mean onset time of 130.67 days and a median onset time of 36 days ([IQR] 11–126 days). Our data showed that the most onset time of azacitidine-related AEs was less than 30 days (n = 3,270, 46.49%). Of note, AEs might still have occurred after half a year for azacitidine treatment, with a proportion of 17.78%.

The main strength of this study is our ability to identify potential adverse events that may have been missed during the clinical trial phase of azacitidine. However, as with previous studies using pharmacovigilance databases, some limitations of the current evaluation should be acknowledged. First, the voluntary nature of reporting to the FAERS and WHO-VigiAccess databases makes it difficult to accurately estimate the incidence and prevalence of adverse events, leading to expected underreporting. Furthermore, the presence of reports in the FAERS and WHO-VigiAccess databases does not imply causation (Maciá-Martínez et al., 2016; Chrétien et al., 2023); therefore, the results of this study only indicate the possible occurrence of adverse events and emphasize the need for vigilance among healthcare professionals such as physicians and pharmacists. Furthermore, various unmeasured confounding factors—including possible drug-drug interactions, pre-existing health conditions, and drug combinations—were left out of our data analysis, which could affect adverse events. Lastly, the disproportionality analysis did not clarify risks or confirm causal relationships, instead providing only an estimation of signal strength that reached statistical significance. Consequently, future prospective clinical trials are essential to substantiate any causal relationships.

## Conclusion

To summarize, this research carried out an in-depth examination of AEs linked to azacitidine, drawing on real-world data from both the FAERS and WHO-VigiAccess databases via disproportionality analysis. The AEs identified in this study were largely consistent with those listed in the product label, while also revealing additional potential AEs, including issues related to death, sepsis, infection, septic shock, respiratory failure, cardiac failure, tumor lysis syndrome, bone marrow failure, and interstitial lung disease, and pericarditis. Moreover, we analyzed the median onset time of these AEs to offer a reference for healthcare providers, aiding in the refinement of medication strategies and addressing safety issues associated with azacitidine. Nevertheless, given the exploratory scope of this study, it is crucial to conduct future prospective clinical trials and gather long-term data to substantiate these results and develop a thorough safety profile for azacitidine.

## Data Availability

The original contributions presented in the study are included in the article/Supplementary Material, further inquiries can be directed to the corresponding author.
