# Supplementary material for: The real-world analysis of adverse events with azacitidine: a pharmacovigilance study based on the FAERS and WHO-VigiAccess databases
Source: Front Pharmacol. 2025 Mar 19;16:1555838. doi: 10.3389/fphar.2025.1555838 (PMC11961983; doi:10.3389/fphar.2025.1555838)
Supplement: Supplementary file 1 [file DataSheet1.docx]

**Table S1 Characteristics of AEs reports for azacitidine in WHO-Vigiaccess database**

| **Characteristics** | **n (%)** |
| --- | --- |
| Sex |  |
| Female | 6987(35.17) |
| Male | 10968(55.21) |
| Unknown | 1912(9.62) |
| Age |  |
| 0 - 27 days | 5(0.03) |
| 28 days to 23 months | 47(0.24) |
| 2 - 11 years | 103(0.52) |
| 12 - 17 years | 114(0.57) |
| 18 - 44 years | 866(4.36) |
| 45 - 64 years | 3550(17.87) |
| 65 - 74 years | 5762(29) |
| 75 years | 5348(26.92) |
| Unknown | 4072(20.5) |
| Continent |  |
| Africa | 52(0.26) |
| Americas | 6123(30.82) |
| Asia | 7013(35.3) |
| Europe | 6020(30.3) |
| Oceania | 659(3.32) |
| Report year |  |
| 1978 | 1(0.01) |
| 1984 | 1(0.01) |
| 1985 | 2(0.01) |
| 1990 | 2(0.01) |
| 1999 | 1(0.01) |
| 2000 | 2(0.01) |
| 2002 | 2(0.01) |
| 2005 | 31(0.16) |
| 2006 | 28(0.14) |
| 2007 | 4(0.02) |
| 2008 | 93(0.47) |
| 2009 | 205(1.03) |
| 2010 | 472(2.38) |
| 2011 | 598(3.01) |
| 2012 | 606(3.05) |
| 2013 | 1131(5.69) |
| 2014 | 1293(6.51) |
| 2015 | 1375(6.92) |
| 2016 | 1334(6.71) |
| 2017 | 944(4.75) |
| 2018 | 1078(5.43) |
| 2019 | 1216(6.12) |
| 2020 | 1055(5.31) |
| 2021 | 1529(7.7) |
| 2022 | 1971(9.92) |
| 2023 | 2072(10.43) |
| 2024 | 2821(14.2) |

**Table S2 Signal strength of ADEs at the System Organ Class (SOC) level for azacitidine in WHO-Vigiaccess database**

| **System Organ Class(SOC)** | **SOC Code** | **Case reports** | **ROR(95% CI)** | **PRR(95% CI)** | **Chi_Square** | **IC(IC025)** | **EBGM(EBGM05)** |
| --- | --- | --- | --- | --- | --- | --- | --- |
| Blood and lymphatic system disorders | 10005329 | 8959 | 10.75(10.51,11.01) | 8.85(8.69,9.02) | 63595.3 | 3.14(3.11) | 8.83(8.62) |
| General disorders and administration site conditions | 10018065 | 6249 | 0.62(0.60,0.63) | 0.67(0.65,0.69) | 1272.40 | -0.58(-0.62) | 0.67(0.65) |
| Infections and infestations | 10021881 | 6075 | 3.65(3.55,3.75) | 3.30(3.22,3.38) | 10139.1 | 1.72(1.68) | 3.30(3.21) |
| Investigations | 10022891 | 4818 | 1.74(1.69,1.79) | 1.66(1.62,1.71) | 1351.80 | 0.73(0.69) | 1.66(1.61) |
| Gastrointestinal disorders | 10017947 | 4247 | 0.92(0.90,0.95) | 0.93(0.90,0.96) | 24.20 | -0.10(-0.15) | 0.93(0.90) |
| Neoplasms benign, malignant and unspecified (incl cysts and polyps) | 10029104 | 2352 | 3.82(3.66,3.98) | 3.67(3.53,3.82) | 4632.34 | 1.88(1.81) | 3.67(3.52) |
| Respiratory, thoracic and mediastinal disorders | 10038738 | 2164 | 1.07(1.03,1.12) | 1.07(1.03,1.12) | 10.61 | 0.10(0.04) | 1.07(1.03) |
| Skin and subcutaneous tissue disorders | 10040785 | 1777 | 0.42(0.40,0.44) | 0.44(0.42,0.46) | 1407.96 | -1.19(-1.26) | 0.44(0.42) |
| Nervous system disorders | 10029205 | 1406 | 0.28(0.26,0.29) | 0.30(0.28,0.31) | 2580.35 | -1.74(-1.82) | 0.30(0.28) |
| Injury, poisoning and procedural complications | 10022117 | 1401 | 0.46(0.44,0.48) | 0.48(0.45,0.50) | 865.31 | -1.07(-1.15) | 0.48(0.45) |
| Cardiac disorders | 10007541 | 1284 | 1.18(1.12,1.25) | 1.18(1.12,1.24) | 35.26 | 0.24(0.15) | 1.18(1.11) |
| Metabolism and nutrition disorders | 10027433 | 1143 | 1.40(1.32,1.48) | 1.39(1.31,1.47) | 127.45 | 0.48(0.39) | 1.39(1.31) |
| Renal and urinary disorders | 10038359 | 803 | 1.09(1.02,1.17) | 1.09(1.02,1.17) | 6.25 | 0.13(0.02) | 1.09(1.02) |
| Vascular disorders | 10047065 | 716 | 0.73(0.67,0.78) | 0.73(0.68,0.79) | 72.76 | -0.45(-0.56) | 0.73(0.68) |
| Musculoskeletal and connective tissue disorders | 10028395 | 700 | 0.28(0.26,0.30) | 0.29(0.27,0.31) | 1290.39 | -1.79(-1.90) | 0.29(0.27) |
| Hepatobiliary disorders | 10019805 | 435 | 1.19(1.08,1.31) | 1.19(1.08,1.30) | 13.02 | 0.25(0.11) | 1.19(1.08) |
| Psychiatric disorders | 10037175 | 373 | 0.16(0.15,0.18) | 0.17(0.15,0.19) | 1572.82 | -2.55(-2.69) | 0.17(0.15) |
| Immune system disorders | 10021428 | 347 | 0.60(0.54,0.66) | 0.60(0.54,0.67) | 93.19 | -0.73(-0.89) | 0.60(0.54) |
| Eye disorders | 10015919 | 156 | 0.19(0.17,0.23) | 0.20(0.17,0.23) | 521.33 | -2.35(-2.57) | 0.20(0.17) |
| Surgical and medical procedures | 10042613 | 133 | 0.36(0.30,0.42) | 0.36(0.30,0.42) | 155.29 | -1.49(-1.73) | 0.36(0.30) |
| Product issues | 10077536 | 90 | 0.22(0.18,0.27) | 0.22(0.18,0.27) | 255.85 | -2.20(-2.49) | 0.22(0.18) |
| Ear and labyrinth disorders | 10013993 | 74 | 0.30(0.24,0.37) | 0.30(0.24,0.37) | 122.62 | -1.74(-2.06) | 0.30(0.24) |
| Social circumstances | 10041244 | 70 | 0.44(0.35,0.56) | 0.44(0.35,0.56) | 49.11 | -1.17(-1.50) | 0.44(0.35) |
| Congenital, familial and genetic disorders | 10010331 | 68 | 0.87(0.69,1.10) | 0.87(0.69,1.10) | 1.32 | -0.20(-0.55) | 0.87(0.69) |
| Reproductive system and breast disorders | 10038604 | 64 | 0.13(0.10,0.17) | 0.13(0.10,0.17) | 374.69 | -2.94(-3.28) | 0.13(0.10) |
| Endocrine disorders | 10014698 | 46 | 0.50(0.37,0.66) | 0.50(0.37,0.66) | 23.38 | -1.01(-1.41) | 0.50(0.37) |
| Pregnancy, puerperium and perinatal conditions | 10036585 | 9 | 0.07(0.04,0.14) | 0.07(0.04,0.14) | 106.54 | -3.78(-4.55) | 0.07(0.04) |

Note:ranked by case reports

**Table S3 Signal strength of adverse events at the Preferred Term(PT) level ranked by EBGM in FAERS database**

| **System Organ Class(SOC)** | **Preferred Term(PT)** | **Case reports** | **ROR (95% CI)** | **PRR (95% CI)** | **Chi Square** | **IC (IC025)** | **EBGM (EBGM05)** |
| --- | --- | --- | --- | --- | --- | --- | --- |
| Neoplasms benign, malignant and unspecified (incl cysts and polyps) | Angioimmunoblastic T-cell lymphoma refractory | 3 | 919.34  (205.75,4107.87) | 919.28  (205.75,4107.36) | 1572.48 | 9.04  (0.22) | 525.73  (117.66) |
| Congenital, familial and genetic disorders | FLT3 gene mutation | 5 | 681.02  (228.22,2032.20) | 680.95  (228.22,2031.81) | 2182.34 | 8.78  (1.17) | 438.11  (146.82) |
| Neoplasms benign, malignant and unspecified (incl cysts and polyps) | Myelodysplastic syndrome transformation | 120 | 356.24  (290.67,436.60) | 355.28  (290.01,435.24) | 32867.2 | 8.11  (6.11) | 275.66  (224.92) |
| Neoplasms benign, malignant and unspecified (incl cysts and polyps) | Juvenile chronic myelomonocytic leukaemia | 9 | 290.36  (140.39,600.50) | 290.30  (140.38,600.31) | 2097.86 | 7.88  (2.26) | 234.90  (113.58) |
| Neoplasms benign, malignant and unspecified (incl cysts and polyps) | Transformation to acute myeloid leukaemia | 91 | 258.12  (205.87,323.65) | 257.60  (205.52,322.86) | 19220.4 | 7.73  (5.68) | 213.03  (169.90) |
| Infections and infestations | Pseudomonal skin infection | 3 | 229.84  (66.97,788.82) | 229.82  (66.97,788.71) | 575.56 | 7.60  (0.40) | 193.69  (56.43) |
| Gastrointestinal disorders | Ulcerative duodenitis | 4 | 204.30  (70.88,588.85) | 204.28  (70.88,588.74) | 693.56 | 7.45  (0.89) | 175.24  (60.80) |
| Neoplasms benign, malignant and unspecified (incl cysts and polyps) | Blastic plasmacytoid dendritic cell neoplasia | 9 | 183.89  (91.26,370.57) | 183.86  (91.25,370.45) | 1423.26 | 7.32  (2.27) | 160.00  (79.40) |
| Hepatobiliary disorders | Portal vein cavernous transformation | 5 | 180.27  (70.50,460.95) | 180.25  (70.50,460.85) | 777.01 | 7.30  (1.28) | 157.27  (61.51) |
| Neoplasms benign, malignant and unspecified (incl cysts and polyps) | Acute myeloid leukaemia refractory | 16 | 173.61  (102.84,293.08) | 173.55  (102.82,292.93) | 2404.46 | 7.25  (3.20) | 152.15  (90.13) |
| Neoplasms benign, malignant and unspecified (incl cysts and polyps) | Acute myeloid leukaemia recurrent | 201 | 170.44  (147.02,197.59) | 169.67  (146.45,196.58) | 29606.3 | 7.22  (6.21) | 149.16  (128.67) |
| Skin and subcutaneous tissue disorders | Neutrophilic panniculitis | 8 | 166.23  (79.43,347.88) | 166.20  (79.42,347.78) | 1156.78 | 7.19  (2.07) | 146.47  (69.99) |
| Investigations | Blast cell count increased | 96 | 147.03  (118.96,181.73) | 146.72  (118.75,181.27) | 12408.5 | 7.03  (5.50) | 131.14  (106.10) |
| Nervous system disorders | Lower motor neurone lesion | 5 | 145.93  (57.73,368.87) | 145.92  (57.73,368.79) | 643.07 | 7.03  (1.28) | 130.50  (51.63) |
| Infections and infestations | Malassezia infection | 4 | 132.52  (47.23,371.81) | 132.51  (47.23,371.75) | 471.13 | 6.90  (0.91) | 119.68  (42.66) |
| Neoplasms benign, malignant and unspecified (incl cysts and polyps) | Chronic myelomonocytic leukaemia | 58 | 131.09  (99.98,171.88) | 130.92  (99.88,171.61) | 6756.35 | 6.89  (4.91) | 118.38  (90.29) |
| Social circumstances | Blood product transfusion dependent | 34 | 114.26  (80.39,162.40) | 114.18  (80.35,162.24) | 3489.26 | 6.71  (4.21) | 104.53  (73.55) |
| Neoplasms benign, malignant and unspecified (incl cysts and polyps) | Acute erythroid leukaemia | 10 | 113.52  (59.38,217.00) | 113.49  (59.38,216.92) | 1020.51 | 6.70  (2.42) | 103.96  (54.38) |
| Neoplasms benign, malignant and unspecified (incl cysts and polyps) | Acute myeloid leukaemia | 1117 | 112.47  (105.72,119.66) | 109.66  (103.22,116.50) | 110418 | 6.65  (6.44) | 100.74  (94.69) |
| Neoplasms benign, malignant and unspecified (incl cysts and polyps) | Leukaemic infiltration pulmonary | 3 | 102.15  (31.46,331.72) | 102.14  (31.46,331.67) | 277.34 | 6.56  (0.44) | 94.36  (29.06) |
| Infections and infestations | Protothecosis | 5 | 100.48  (40.37,250.07) | 100.47  (40.37,250.02) | 455.09 | 6.54  (1.28) | 92.93  (37.34) |
| Infections and infestations | Gastroenteritis astroviral | 4 | 100.07  (36.11,277.30) | 100.06  (36.11,277.25) | 362.67 | 6.53  (0.91) | 92.58  (33.41) |
| General disorders and administration site conditions | Injection site vasculitis | 4 | 96.14  (34.75,266.02) | 96.13  (34.75,265.98) | 349.19 | 6.48  (0.91) | 89.21  (32.24) |
| Infections and infestations | Emphysematous cholecystitis | 3 | 94.29  (29.14,305.13) | 94.28  (29.14,305.09) | 257.11 | 6.45  (0.44) | 87.62  (27.08) |
| General disorders and administration site conditions | Administration site induration | 5 | 91.48  (36.87,226.97) | 91.47  (36.87,226.93) | 416.34 | 6.41  (1.28) | 85.19  (34.33) |
| Vascular disorders | Venous aneurysm | 3 | 89.69  (27.77,289.66) | 89.69  (27.77,289.61) | 245.15 | 6.39  (0.44) | 83.64  (25.90) |
| Infections and infestations | Sphingomonas paucimobilis infection | 3 | 89.69  (27.77,289.66) | 89.69  (27.77,289.61) | 245.15 | 6.39  (0.44) | 83.64  (25.90) |
| Neoplasms benign, malignant and unspecified (incl cysts and polyps) | Leukaemia cutis | 8 | 85.28  (41.65,174.63) | 85.27  (41.64,174.58) | 622.89 | 6.32  (2.03) | 79.79  (38.96) |
| Surgical and medical procedures | Allogenic stem cell transplantation | 7 | 78.73  (36.66,169.06) | 78.71  (36.66,169.02) | 504.68 | 6.21  (1.81) | 74.03  (34.47) |
| Infections and infestations | Disseminated trichosporonosis | 3 | 78.24  (24.35,251.39) | 78.24  (24.35,251.35) | 215.02 | 6.20  (0.44) | 73.60  (22.91) |
| Neoplasms benign, malignant and unspecified (incl cysts and polyps) | Acute megakaryocytic leukaemia | 3 | 73.55  (22.94,235.81) | 73.54  (22.94,235.78) | 202.52 | 6.12  (0.44) | 69.44  (21.66) |
| Blood and lymphatic system disorders | Leukostasis syndrome | 8 | 72.11  (35.34,147.14) | 72.10  (35.34,147.09) | 529.75 | 6.09  (2.02) | 68.15  (33.40) |
| Infections and infestations | Application site abscess | 3 | 72.11  (22.50,231.04) | 72.10  (22.50,231.00) | 198.66 | 6.09  (0.44) | 68.15  (21.27) |
| Infections and infestations | Myocardiac abscess | 3 | 72.11  (22.50,231.04) | 72.10  (22.50,231.00) | 198.66 | 6.09  (0.44) | 68.15  (21.27) |
| Infections and infestations | Bacillus bacteraemia | 5 | 71.27  (28.93,175.60) | 71.26  (28.93,175.56) | 327.35 | 6.07  (1.26) | 67.40  (27.36) |
| Neoplasms benign, malignant and unspecified (incl cysts and polyps) | Myelodysplastic syndrome with excess blasts | 19 | 65.08  (41.02,103.25) | 65.05  (41.01,103.18) | 1137.89 | 5.95  (3.27) | 61.82  (38.97) |
| Injury, poisoning and procedural complications | Refractoriness to platelet transfusion | 5 | 63.85  (25.98,156.90) | 63.84  (25.98,156.87) | 293.96 | 5.92  (1.26) | 60.73  (24.71) |
| Infections and infestations | Hepatic infection fungal | 5 | 62.54  (25.46,153.63) | 62.54  (25.46,153.59) | 288.06 | 5.90  (1.26) | 59.55  (24.24) |
| Neoplasms benign, malignant and unspecified (incl cysts and polyps) | Chloroma | 18 | 60.47  (37.67,97.07) | 60.45  (37.66,97.02) | 1002.87 | 5.85  (3.18) | 57.65  (35.91) |
| Infections and infestations | Geotrichum infection | 8 | 59.44  (29.23,120.85) | 59.43  (29.23,120.82) | 438.31 | 5.83  (1.99) | 56.73  (27.90) |
| Investigations | Blast cell count decreased | 3 | 59.31  (18.62,188.96) | 59.31  (18.62,188.93) | 164.04 | 5.82  (0.44) | 56.62  (17.77) |
| Neoplasms benign, malignant and unspecified (incl cysts and polyps) | Minimal residual disease | 15 | 56.24  (33.52,94.38) | 56.22  (33.51,94.33) | 777.96 | 5.75  (2.91) | 53.80  (32.06) |
| Investigations | Karyotype analysis abnormal | 4 | 55.09  (20.23,150.03) | 55.09  (20.23,150.00) | 203.29 | 5.72  (0.89) | 52.76  (19.37) |
| Congenital, familial and genetic disorders | Chimerism | 3 | 54.89  (17.26,174.51) | 54.88  (17.26,174.48) | 151.90 | 5.72  (0.44) | 52.57  (16.54) |
| Cardiac disorders | Agonal rhythm | 4 | 53.88  (19.80,146.66) | 53.88  (19.80,146.64) | 198.84 | 5.69  (0.89) | 51.65  (18.98) |
| Endocrine disorders | Euthyroid sick syndrome | 3 | 53.30  (16.77,169.33) | 53.29  (16.77,169.30) | 147.52 | 5.68  (0.43) | 51.11  (16.09) |
| Gastrointestinal disorders | Ulcerative gastritis | 4 | 52.72  (19.38,143.44) | 52.72  (19.38,143.42) | 194.58 | 5.66  (0.89) | 50.59  (18.59) |
| Neoplasms benign, malignant and unspecified (incl cysts and polyps) | Myelodysplastic syndrome | 502 | 49.48  (45.24,54.12) | 48.93  (44.78,53.47) | 22670.1 | 5.56  (5.30) | 47.09  (43.05) |
| Blood and lymphatic system disorders | Bone marrow necrosis | 8 | 48.79  (24.07,98.92) | 48.78  (24.07,98.89) | 360.11 | 5.55  (1.96) | 46.96  (23.16) |
| Neoplasms benign, malignant and unspecified (incl cysts and polyps) | Leukaemic infiltration | 4 | 46.26  (17.04,125.53) | 46.25  (17.04,125.51) | 170.66 | 5.48  (0.88) | 44.61  (16.44) |
| Nervous system disorders | Autoimmune encephalopathy | 3 | 45.97  (14.52,145.56) | 45.96  (14.52,145.54) | 127.19 | 5.47  (0.43) | 44.34  (14.00) |
| Infections and infestations | Acinetobacter bacteraemia | 6 | 45.12  (19.98,101.92) | 45.12  (19.98,101.90) | 249.65 | 5.44  (1.51) | 43.55  (19.28) |
| Skin and subcutaneous tissue disorders | Acute febrile neutrophilic dermatosis | 69 | 44.82  (35.24,57.00) | 44.75  (35.20,56.89) | 2847.31 | 5.43  (4.40) | 43.21  (33.97) |
| Infections and infestations | Pneumonia fungal | 131 | 42.69  (35.86,50.83) | 42.57  (35.77,50.66) | 5139.41 | 5.36  (4.72) | 41.17  (34.58) |
| Neoplasms benign, malignant and unspecified (incl cysts and polyps) | Differentiation syndrome | 29 | 41.31  (28.53,59.82) | 41.28  (28.52,59.76) | 1102.81 | 5.32  (3.58) | 39.97  (27.61) |
| Investigations | Bacillus test positive | 3 | 40.86  (12.93,129.09) | 40.86  (12.93,129.07) | 112.88 | 5.31  (0.42) | 39.57  (12.53) |
| Investigations | Aspiration bone marrow abnormal | 4 | 38.91  (14.38,105.30) | 38.91  (14.38,105.29) | 143.20 | 5.24  (0.86) | 37.74  (13.95) |
| Infections and infestations | Adenovirus reactivation | 3 | 38.71  (12.26,122.17) | 38.71  (12.26,122.16) | 106.82 | 5.23  (0.42) | 37.55  (11.90) |
| Infections and infestations | Anorectal infection | 7 | 38.65  (18.21,82.03) | 38.65  (18.21,82.01) | 248.87 | 5.23  (1.71) | 37.50  (17.67) |
| Neoplasms benign, malignant and unspecified (incl cysts and polyps) | Central nervous system leukaemia | 10 | 37.72  (20.10,70.78) | 37.71  (20.10,70.76) | 346.74 | 5.19  (2.23) | 36.62  (19.52) |
| Immune system disorders | Graft versus host disease in liver | 18 | 37.66  (23.56,60.21) | 37.65  (23.56,60.17) | 623.04 | 5.19  (3.00) | 36.56  (22.87) |
| Blood and lymphatic system disorders | Febrile neutropenia | 1622 | 37.36  (35.53,39.29) | 36.03  (34.32,37.82) | 53717.2 | 5.13  (5.03) | 35.03  (33.31) |
| Infections and infestations | Bacillus infection | 16 | 36.88  (22.42,60.64) | 36.86  (22.42,60.61) | 541.95 | 5.16  (2.84) | 35.82  (21.78) |
| Injury, poisoning and procedural complications | Transfusion reaction | 21 | 34.71  (22.49,53.56) | 34.69  (22.48,53.52) | 668.19 | 5.08  (3.14) | 33.76  (21.88) |
| Infections and infestations | Infective myositis | 4 | 34.29  (12.69,92.62) | 34.29  (12.69,92.60) | 125.74 | 5.06  (0.85) | 33.38  (12.36) |
| Infections and infestations | Aeromonas infection | 5 | 33.68  (13.85,81.89) | 33.67  (13.85,81.87) | 154.28 | 5.04  (1.18) | 32.80  (13.49) |
| Blood and lymphatic system disorders | Cytopenia | 239 | 33.03  (29.04,37.57) | 32.86  (28.91,37.35) | 7191.18 | 5.00  (4.64) | 32.03  (28.16) |
| Neoplasms benign, malignant and unspecified (incl cysts and polyps) | Leukaemic infiltration extramedullary | 5 | 32.78  (13.48,79.67) | 32.77  (13.48,79.66) | 150.01 | 5.00  (1.18) | 31.95  (13.14) |
| Infections and infestations | Periorbital cellulitis | 12 | 32.48  (18.31,57.62) | 32.47  (18.30,57.60) | 356.56 | 4.98  (2.42) | 31.66  (17.84) |
| Infections and infestations | Viral sepsis | 3 | 32.26  (10.25,101.51) | 32.26  (10.25,101.50) | 88.53 | 4.98  (0.40) | 31.45  (10.00) |
| General disorders and administration site conditions | Administration site erythema | 6 | 31.70  (14.10,71.30) | 31.70  (14.10,71.29) | 173.89 | 4.95  (1.44) | 30.93  (13.75) |
| Infections and infestations | Pulmonary sepsis | 32 | 31.40  (22.10,44.60) | 31.38  (22.09,44.56) | 917.65 | 4.94  (3.50) | 30.62  (21.56) |
| Skin and subcutaneous tissue disorders | Neutrophilic dermatosis | 8 | 31.13  (15.43,62.81) | 31.13  (15.43,62.79) | 227.51 | 4.93  (1.86) | 30.38  (15.06) |
| Gastrointestinal disorders | Gastric volvulus | 3 | 30.90  (9.83,97.19) | 30.90  (9.83,97.17) | 84.66 | 4.91  (0.40) | 30.16  (9.59) |
| Infections and infestations | Clostridium bacteraemia | 4 | 29.54  (10.96,79.63) | 29.54  (10.96,79.62) | 107.68 | 4.85  (0.82) | 28.86  (10.71) |
| Infections and infestations | Bronchopulmonary aspergillosis | 154 | 29.27  (24.94,34.35) | 29.17  (24.87,34.22) | 4093.22 | 4.83  (4.36) | 28.52  (24.30) |
| Infections and infestations | Neutropenic infection | 21 | 29.06  (18.85,44.81) | 29.05  (18.85,44.78) | 555.65 | 4.83  (3.04) | 28.40  (18.42) |
| Infections and infestations | Pseudomonal bacteraemia | 16 | 28.51  (17.37,46.81) | 28.50  (17.37,46.79) | 414.99 | 4.80  (2.72) | 27.88  (16.98) |
| Investigations | Haemoglobin | 4 | 28.18  (10.46,75.93) | 28.18  (10.46,75.92) | 102.49 | 4.78  (0.82) | 27.57  (10.23) |
| Infections and infestations | Hepatosplenic candidiasis | 3 | 28.07  (8.94,88.17) | 28.07  (8.94,88.16) | 76.56 | 4.78  (0.39) | 27.46  (8.74) |
| Infections and infestations | Soft tissue infection | 36 | 28.04  (20.15,39.02) | 28.02  (20.14,38.98) | 916.93 | 4.78  (3.52) | 27.41  (19.70) |
| Infections and infestations | Sinusitis aspergillus | 3 | 27.65  (8.80,86.83) | 27.65  (8.80,86.82) | 75.35 | 4.76  (0.38) | 27.06  (8.62) |
| Infections and infestations | Fusarium infection | 12 | 27.24  (15.37,48.28) | 27.24  (15.37,48.26) | 296.70 | 4.74  (2.35) | 26.67  (15.05) |
| Investigations | Granulocytes abnormal | 4 | 27.09  (10.06,72.96) | 27.09  (10.06,72.95) | 98.32 | 4.73  (0.81) | 26.52  (9.85) |
| Neoplasms benign, malignant and unspecified (incl cysts and polyps) | Leukaemia recurrent | 33 | 26.65  (18.87,37.63) | 26.63  (18.86,37.59) | 796.69 | 4.71  (3.41) | 26.08  (18.47) |
| Infections and infestations | Lower respiratory tract infection fungal | 8 | 26.51  (13.16,53.40) | 26.50  (13.16,53.39) | 192.16 | 4.70  (1.81) | 25.96  (12.89) |
| Infections and infestations | Splenic abscess | 7 | 26.32  (12.45,55.66) | 26.32  (12.45,55.64) | 166.91 | 4.69  (1.62) | 25.79  (12.20) |
| Vascular disorders | Angiodysplasia | 7 | 26.24  (12.41,55.49) | 26.24  (12.41,55.47) | 166.37 | 4.68  (1.62) | 25.71  (12.16) |
| Musculoskeletal and connective tissue disorders | Gouty tophus | 4 | 25.67  (9.54,69.11) | 25.67  (9.54,69.09) | 92.89 | 4.65  (0.80) | 25.16  (9.35) |
| Infections and infestations | Enterobacter bacteraemia | 5 | 25.22  (10.40,61.15) | 25.22  (10.40,61.14) | 113.96 | 4.63  (1.13) | 24.73  (10.20) |
| Infections and infestations | Cystitis klebsiella | 3 | 25.19  (8.03,79.01) | 25.19  (8.03,79.00) | 68.27 | 4.63  (0.37) | 24.70  (7.87) |
| Product issues | Product reconstitution quality issue | 18 | 24.36  (15.28,38.85) | 24.35  (15.27,38.82) | 395.23 | 4.58  (2.77) | 23.90  (14.99) |
| Neoplasms benign, malignant and unspecified (incl cysts and polyps) | Acute leukaemia | 28 | 23.47  (16.15,34.12) | 23.46  (16.14,34.09) | 590.74 | 4.53  (3.17) | 23.04  (15.85) |
| Metabolism and nutrition disorders | Tumour lysis syndrome | 137 | 23.38  (19.74,27.70) | 23.31  (19.69,27.60) | 2871.50 | 4.52  (4.06) | 22.90  (19.33) |
| Neoplasms benign, malignant and unspecified (incl cysts and polyps) | Acute myelomonocytic leukaemia | 4 | 23.13  (8.60,62.20) | 23.13  (8.60,62.19) | 83.11 | 4.51  (0.78) | 22.72  (8.45) |
| Infections and infestations | Escherichia sepsis | 48 | 22.95  (17.25,30.54) | 22.93  (17.24,30.50) | 988.21 | 4.49  (3.55) | 22.53  (16.93) |
| Infections and infestations | Klebsiella sepsis | 18 | 22.82  (14.32,36.39) | 22.82  (14.31,36.37) | 368.62 | 4.49  (2.73) | 22.42  (14.06) |
| Infections and infestations | Anal infection | 4 | 22.49  (8.37,60.47) | 22.49  (8.37,60.46) | 80.66 | 4.47  (0.78) | 22.10  (8.22) |
| Infections and infestations | Neutropenic sepsis | 115 | 22.12  (18.39,26.61) | 22.07  (18.36,26.53) | 2272.53 | 4.44  (3.93) | 21.70  (18.04) |
| Infections and infestations | Respiratory moniliasis | 4 | 22.09  (8.22,59.37) | 22.08  (8.22,59.36) | 79.10 | 4.44  (0.77) | 21.71  (8.08) |
| Gastrointestinal disorders | Neutropenic colitis | 30 | 21.98  (15.32,31.54) | 21.97  (15.31,31.51) | 589.80 | 4.43  (3.17) | 21.60  (15.05) |
| Infections and infestations | Candida pneumonia | 5 | 21.97  (9.07,53.20) | 21.97  (9.07,53.19) | 98.30 | 4.43  (1.09) | 21.60  (8.92) |
| Metabolism and nutrition disorders | Iron overload | 17 | 21.96  (13.60,35.49) | 21.96  (13.59,35.47) | 334.06 | 4.43  (2.64) | 21.59  (13.36) |
| Investigations | Gram stain positive | 3 | 21.38  (6.83,66.95) | 21.38  (6.83,66.94) | 57.28 | 4.39  (0.35) | 21.03  (6.72) |
| General disorders and administration site conditions | Application site haematoma | 3 | 21.38  (6.83,66.95) | 21.38  (6.83,66.94) | 57.28 | 4.39  (0.35) | 21.03  (6.72) |
| General disorders and administration site conditions | Injection site plaque | 3 | 21.26  (6.79,66.56) | 21.25  (6.79,66.55) | 56.92 | 4.39  (0.35) | 20.91  (6.68) |
| Gastrointestinal disorders | Abdominal wall haemorrhage | 4 | 21.13  (7.87,56.79) | 21.13  (7.87,56.78) | 75.42 | 4.38  (0.76) | 20.79  (7.74) |
| Infections and infestations | Febrile infection | 16 | 20.56  (12.55,33.71) | 20.56  (12.54,33.69) | 292.78 | 4.34  (2.54) | 20.23  (12.34) |
| Infections and infestations | Clostridial sepsis | 5 | 20.50  (8.47,49.61) | 20.50  (8.47,49.60) | 91.20 | 4.33  (1.08) | 20.18  (8.34) |
| Infections and infestations | Epstein-Barr virus infection reactivation | 14 | 20.48  (12.08,34.74) | 20.48  (12.08,34.72) | 255.11 | 4.33  (2.39) | 20.16  (11.89) |
| Respiratory, thoracic and mediastinal disorders | Organising pneumonia | 73 | 20.47  (16.24,25.80) | 20.43  (16.22,25.75) | 1327.17 | 4.33  (3.66) | 20.11  (15.96) |
| General disorders and administration site conditions | Administration site reaction | 5 | 19.77  (8.17,47.84) | 19.77  (8.17,47.83) | 87.69 | 4.28  (1.07) | 19.47  (8.05) |
| Blood and lymphatic system disorders | Febrile bone marrow aplasia | 55 | 19.41  (14.87,25.33) | 19.38  (14.85,25.29) | 944.03 | 4.26  (3.46) | 19.10  (14.63) |
| Cardiac disorders | Myopericarditis | 7 | 19.03  (9.02,40.14) | 19.02  (9.02,40.13) | 117.71 | 4.23  (1.51) | 18.75  (8.89) |
| Infections and infestations | Pseudomonal sepsis | 23 | 18.93  (12.54,28.58) | 18.92  (12.53,28.56) | 384.46 | 4.22  (2.83) | 18.65  (12.35) |
| Infections and infestations | Human herpesvirus 6 infection reactivation | 4 | 18.79  (7.00,50.43) | 18.78  (7.00,50.42) | 66.34 | 4.21  (0.74) | 18.52  (6.90) |
| Surgical and medical procedures | Pulmonary resection | 3 | 18.30  (5.85,57.21) | 18.29  (5.85,57.20) | 48.33 | 4.17  (0.32) | 18.04  (5.77) |
| Injury, poisoning and procedural complications | Complications of transplant surgery | 7 | 18.10  (8.58,38.18) | 18.10  (8.58,38.17) | 111.45 | 4.16  (1.49) | 17.85  (8.46) |
| Infections and infestations | Scedosporium infection | 8 | 18.09  (9.00,36.37) | 18.09  (9.00,36.36) | 127.30 | 4.16  (1.66) | 17.84  (8.88) |
| Infections and infestations | Systemic candida | 29 | 18.07  (12.52,26.08) | 18.06  (12.52,26.06) | 460.62 | 4.15  (2.98) | 17.81  (12.34) |
| Infections and infestations | Sinusitis fungal | 9 | 17.63  (9.13,34.04) | 17.62  (9.13,34.02) | 139.11 | 4.12  (1.80) | 17.39  (9.00) |
| Blood and lymphatic system disorders | Hyperleukocytosis | 10 | 17.51  (9.38,32.70) | 17.51  (9.38,32.69) | 153.48 | 4.11  (1.92) | 17.28  (9.25) |
| Infections and infestations | Respiratory tract infection fungal | 7 | 17.41  (8.25,36.71) | 17.40  (8.25,36.70) | 106.71 | 4.10  (1.48) | 17.17  (8.14) |
| Infections and infestations | Enterocolitis infectious | 12 | 17.15  (9.70,30.32) | 17.14  (9.70,30.30) | 179.90 | 4.08  (2.12) | 16.92  (9.57) |
| Infections and infestations | Enteritis infectious | 21 | 17.13  (11.14,26.36) | 17.13  (11.13,26.34) | 314.48 | 4.08  (2.67) | 16.90  (10.99) |
| Neoplasms benign, malignant and unspecified (incl cysts and polyps) | Myelofibrosis | 39 | 17.12  (12.48,23.49) | 17.11  (12.47,23.46) | 583.42 | 4.08  (3.14) | 16.89  (12.31) |
| Immune system disorders | Acute graft versus host disease in liver | 9 | 17.00  (8.81,32.82) | 17.00  (8.80,32.81) | 133.65 | 4.07  (1.78) | 16.78  (8.69) |
| Immune system disorders | Chronic graft versus host disease in liver | 4 | 16.97  (6.32,45.51) | 16.96  (6.32,45.51) | 59.27 | 4.07  (0.71) | 16.75  (6.24) |
| Blood and lymphatic system disorders | Splenic infarction | 21 | 16.92  (11.00,26.03) | 16.91  (11.00,26.01) | 310.12 | 4.06  (2.66) | 16.70  (10.85) |
| Infections and infestations | Pneumonia respiratory syncytial viral | 9 | 16.90  (8.75,32.62) | 16.89  (8.75,32.61) | 132.74 | 4.06  (1.78) | 16.68  (8.64) |
| Congenital, familial and genetic disorders | Congenital aplasia | 29 | 16.89  (11.71,24.37) | 16.88  (11.70,24.35) | 427.32 | 4.06  (2.92) | 16.66  (11.55) |
| Investigations | Enterococcus test positive | 8 | 16.85  (8.39,33.86) | 16.85  (8.39,33.85) | 117.64 | 4.06  (1.63) | 16.63  (8.28) |
| Blood and lymphatic system disorders | Haematotoxicity | 100 | 16.82  (13.81,20.50) | 16.79  (13.78,20.45) | 1464.91 | 4.05  (3.55) | 16.57  (13.60) |
| Investigations | Enterobacter test positive | 3 | 16.13  (5.16,50.38) | 16.13  (5.16,50.38) | 42.02 | 3.99  (0.29) | 15.93  (5.10) |
| Blood and lymphatic system disorders | Monocytosis | 7 | 16.07  (7.62,33.87) | 16.07  (7.62,33.86) | 97.63 | 3.99  (1.44) | 15.87  (7.53) |
| Immune system disorders | Graft versus host disease | 83 | 15.94  (12.83,19.80) | 15.91  (12.82,19.75) | 1145.08 | 3.97  (3.42) | 15.72  (12.66) |
| Immune system disorders | Graft versus host disease in skin | 24 | 15.61  (10.43,23.34) | 15.60  (10.43,23.33) | 323.77 | 3.95  (2.71) | 15.41  (10.30) |
| Skin and subcutaneous tissue disorders | Pyoderma gangrenosum | 36 | 15.58  (11.21,21.65) | 15.57  (11.21,21.63) | 484.70 | 3.94  (2.99) | 15.39  (11.07) |
| Neoplasms benign, malignant and unspecified (incl cysts and polyps) | Myeloproliferative neoplasm | 10 | 15.56  (8.34,29.03) | 15.55  (8.34,29.02) | 134.48 | 3.94  (1.86) | 15.37  (8.24) |
| Infections and infestations | Escherichia bacteraemia | 24 | 15.51  (10.37,23.20) | 15.50  (10.36,23.18) | 321.47 | 3.94  (2.70) | 15.32  (10.24) |
| Infections and infestations | Stenotrophomonas infection | 14 | 15.48  (9.14,26.22) | 15.47  (9.13,26.21) | 187.17 | 3.93  (2.22) | 15.29  (9.03) |
| Reproductive system and breast disorders | Vulval ulceration | 3 | 15.45  (4.95,48.25) | 15.45  (4.95,48.24) | 40.04 | 3.93  (0.29) | 15.27  (4.89) |
| Infections and infestations | Infective aneurysm | 6 | 15.32  (6.85,34.28) | 15.32  (6.85,34.27) | 79.33 | 3.92  (1.23) | 15.14  (6.77) |
| Infections and infestations | Pulmonary mucormycosis | 4 | 15.18  (5.66,40.69) | 15.18  (5.66,40.69) | 52.33 | 3.91  (0.68) | 15.01  (5.60) |
| Injury, poisoning and procedural complications | Complications of bone marrow transplant | 4 | 15.13  (5.65,40.57) | 15.13  (5.65,40.56) | 52.15 | 3.90  (0.68) | 14.96  (5.58) |
| Gastrointestinal disorders | Gastric varices | 5 | 15.10  (6.25,36.47) | 15.09  (6.25,36.46) | 65.01 | 3.90  (0.98) | 14.92  (6.18) |
| Respiratory, thoracic and mediastinal disorders | Lung opacity | 22 | 15.03  (9.87,22.89) | 15.02  (9.87,22.87) | 284.48 | 3.89  (2.61) | 14.85  (9.75) |
| Infections and infestations | Enterobacter sepsis | 4 | 14.99  (5.59,40.19) | 14.99  (5.59,40.19) | 51.61 | 3.89  (0.68) | 14.82  (5.53) |
| Infections and infestations | Herpes simplex reactivation | 3 | 14.77  (4.73,46.11) | 14.77  (4.73,46.10) | 38.05 | 3.87  (0.28) | 14.60  (4.68) |
| Immune system disorders | Graft versus host disease in gastrointestinal tract | 23 | 14.71  (9.75,22.20) | 14.71  (9.75,22.18) | 290.33 | 3.86  (2.62) | 14.54  (9.64) |
| Cardiac disorders | Pericarditis constrictive | 4 | 14.64  (5.46,39.23) | 14.64  (5.46,39.22) | 50.22 | 3.86  (0.67) | 14.47  (5.40) |
| Nervous system disorders | Central nervous system haemorrhage | 5 | 14.63  (6.06,35.33) | 14.63  (6.06,35.32) | 62.73 | 3.85  (0.97) | 14.47  (5.99) |
| General disorders and administration site conditions | Injection site exfoliation | 7 | 14.62  (6.94,30.80) | 14.62  (6.94,30.79) | 87.75 | 3.85  (1.40) | 14.46  (6.86) |
| Neoplasms benign, malignant and unspecified (incl cysts and polyps) | Malignant neoplasm of unknown primary site | 4 | 14.55  (5.43,38.99) | 14.55  (5.43,38.99) | 49.88 | 3.85  (0.67) | 14.39  (5.37) |
| Infections and infestations | Necrotising fasciitis | 35 | 14.50  (10.39,20.24) | 14.49  (10.38,20.22) | 434.39 | 3.84  (2.90) | 14.33  (10.27) |
| Immune system disorders | Acute graft versus host disease | 48 | 14.48  (10.90,19.26) | 14.47  (10.89,19.23) | 594.89 | 3.84  (3.08) | 14.31  (10.77) |
| Immune system disorders | Chronic graft versus host disease | 36 | 14.11  (10.16,19.60) | 14.10  (10.15,19.58) | 433.27 | 3.80  (2.89) | 13.95  (10.04) |
| Infections and infestations | Septic shock | 415 | 14.01  (12.71,15.44) | 13.89  (12.62,15.29) | 4912.12 | 3.78  (3.59) | 13.75  (12.47) |
| Infections and infestations | Eye infection staphylococcal | 3 | 13.93  (4.46,43.47) | 13.93  (4.46,43.46) | 35.60 | 3.78  (0.26) | 13.78  (4.42) |
| Musculoskeletal and connective tissue disorders | Arthritis reactive | 8 | 13.75  (6.85,27.61) | 13.75  (6.85,27.61) | 93.55 | 3.77  (1.53) | 13.61  (6.78) |
| Hepatobiliary disorders | Non-cirrhotic portal hypertension | 3 | 13.67  (4.38,42.66) | 13.67  (4.38,42.65) | 34.84 | 3.76  (0.26) | 13.53  (4.34) |
| Infections and infestations | Bacteraemia | 108 | 13.42  (11.10,16.23) | 13.39  (11.08,16.18) | 1225.06 | 3.73  (3.30) | 13.26  (10.96) |
| Infections and infestations | Pneumonia klebsiella | 17 | 13.27  (8.23,21.40) | 13.26  (8.22,21.39) | 190.70 | 3.72  (2.29) | 13.13  (8.14) |
| Respiratory, thoracic and mediastinal disorders | Alveolar proteinosis | 5 | 13.18  (5.46,31.82) | 13.18  (5.46,31.81) | 55.68 | 3.71  (0.93) | 13.05  (5.41) |
| Investigations | Fibrin degradation products increased | 3 | 13.18  (4.23,41.12) | 13.18  (4.23,41.11) | 33.41 | 3.71  (0.25) | 13.05  (4.18) |
| Infections and infestations | Dermo-hypodermitis | 5 | 13.07  (5.41,31.55) | 13.07  (5.41,31.54) | 55.13 | 3.69  (0.93) | 12.94  (5.36) |
| Infections and infestations | Systemic mycosis | 10 | 13.04  (6.99,24.32) | 13.04  (6.99,24.31) | 109.99 | 3.69  (1.76) | 12.91  (6.92) |
| Neoplasms benign, malignant and unspecified (incl cysts and polyps) | Leukaemia | 87 | 12.95  (10.48,15.99) | 12.92  (10.46,15.96) | 947.15 | 3.68  (3.19) | 12.80  (10.36) |
| Infections and infestations | Enterococcal infection | 43 | 12.82  (9.49,17.32) | 12.81  (9.49,17.30) | 463.43 | 3.67  (2.89) | 12.69  (9.39) |
| Neoplasms benign, malignant and unspecified (incl cysts and polyps) | Primary myelofibrosis | 3 | 12.81  (4.11,39.97) | 12.81  (4.11,39.96) | 32.33 | 3.67  (0.24) | 12.69  (4.07) |
| Infections and infestations | Pneumonia haemophilus | 4 | 12.74  (4.76,34.11) | 12.73  (4.76,34.10) | 42.81 | 3.66  (0.62) | 12.61  (4.71) |
| Infections and infestations | Metapneumovirus infection | 6 | 12.62  (5.64,28.20) | 12.61  (5.64,28.19) | 63.51 | 3.64  (1.14) | 12.50  (5.59) |
| Infections and infestations | Pneumonia necrotising | 5 | 12.56  (5.20,30.31) | 12.56  (5.20,30.31) | 52.65 | 3.64  (0.91) | 12.44  (5.15) |
| General disorders and administration site conditions | Soft tissue inflammation | 3 | 12.42  (3.98,38.74) | 12.42  (3.98,38.74) | 31.19 | 3.62  (0.23) | 12.31  (3.95) |
| Infections and infestations | Endocarditis bacterial | 7 | 12.29  (5.84,25.89) | 12.29  (5.84,25.88) | 71.89 | 3.61  (1.32) | 12.18  (5.78) |
| General disorders and administration site conditions | Application site oedema | 3 | 12.26  (3.93,38.22) | 12.26  (3.93,38.22) | 30.71 | 3.60  (0.23) | 12.15  (3.89) |
| Infections and infestations | Aspergillus infection | 65 | 12.22  (9.57,15.61) | 12.21  (9.56,15.58) | 662.22 | 3.60  (3.01) | 12.10  (9.47) |
| Musculoskeletal and connective tissue disorders | Soft tissue necrosis | 6 | 12.22  (5.47,27.31) | 12.22  (5.47,27.30) | 61.18 | 3.60  (1.13) | 12.11  (5.42) |
| Immune system disorders | Graft versus host disease in lung | 4 | 12.20  (4.56,32.66) | 12.20  (4.56,32.65) | 40.71 | 3.60  (0.61) | 12.09  (4.51) |
| Neoplasms benign, malignant and unspecified (incl cysts and polyps) | Myelodysplastic syndrome with single lineage dysplasia | 4 | 12.14  (4.53,32.50) | 12.14  (4.53,32.49) | 40.47 | 3.59  (0.61) | 12.03  (4.49) |
| Infections and infestations | Cellulitis orbital | 5 | 12.07  (5.00,29.11) | 12.06  (5.00,29.11) | 50.24 | 3.58  (0.90) | 11.96  (4.95) |
| Infections and infestations | Pseudomembranous colitis | 20 | 11.98  (7.71,18.61) | 11.98  (7.71,18.60) | 199.24 | 3.57  (2.33) | 11.87  (7.64) |
| Cardiac disorders | Pericarditis | 114 | 11.88  (9.88,14.29) | 11.85  (9.86,14.25) | 1121.99 | 3.55  (3.15) | 11.75  (9.77) |
| Hepatobiliary disorders | Hepatobiliary disease | 5 | 11.88  (4.92,28.66) | 11.88  (4.92,28.66) | 49.33 | 3.56  (0.89) | 11.77  (4.88) |
| Infections and infestations | Enterococcal sepsis | 7 | 11.87  (5.64,24.99) | 11.87  (5.64,24.98) | 68.99 | 3.56  (1.30) | 11.76  (5.59) |
| Blood and lymphatic system disorders | Pancytopenia | 456 | 11.72  (10.68,12.86) | 11.61  (10.59,12.73) | 4384.77 | 3.53  (3.36) | 11.51  (10.49) |
| Investigations | Neutrophil count decreased | 324 | 11.70  (10.48,13.05) | 11.62  (10.42,12.96) | 3116.92 | 3.53  (3.32) | 11.52  (10.32) |
| Infections and infestations | Cystitis escherichia | 3 | 11.67  (3.74,36.39) | 11.67  (3.74,36.39) | 29.00 | 3.53  (0.21) | 11.57  (3.71) |
| Infections and infestations | Bacterial sepsis | 27 | 11.67  (7.99,17.05) | 11.66  (7.98,17.03) | 260.70 | 3.53  (2.52) | 11.56  (7.91) |
| Immune system disorders | Acute graft versus host disease in intestine | 11 | 11.63  (6.42,21.05) | 11.62  (6.42,21.05) | 105.80 | 3.53  (1.78) | 11.52  (6.36) |
| Infections and infestations | Fungal sepsis | 7 | 11.58  (5.50,24.38) | 11.58  (5.50,24.37) | 67.02 | 3.52  (1.29) | 11.48  (5.45) |
| Infections and infestations | Muscle abscess | 5 | 11.54  (4.78,27.85) | 11.54  (4.78,27.84) | 47.69 | 3.52  (0.88) | 11.44  (4.74) |
| Infections and infestations | Staphylococcal sepsis | 42 | 11.44  (8.44,15.51) | 11.43  (8.44,15.49) | 396.17 | 3.50  (2.75) | 11.34  (8.36) |
| Infections and infestations | Enterococcal bacteraemia | 8 | 11.42  (5.69,22.90) | 11.42  (5.69,22.90) | 75.32 | 3.50  (1.43) | 11.32  (5.64) |
| Infections and infestations | Corynebacterium infection | 4 | 11.35  (4.24,30.38) | 11.35  (4.24,30.38) | 37.40 | 3.49  (0.58) | 11.25  (4.20) |
| Immune system disorders | Acute graft versus host disease in skin | 21 | 11.27  (7.34,17.33) | 11.27  (7.33,17.32) | 194.74 | 3.48  (2.32) | 11.18  (7.27) |
| Infections and infestations | Urinary tract infection enterococcal | 8 | 11.23  (5.60,22.54) | 11.23  (5.60,22.53) | 73.89 | 3.48  (1.42) | 11.14  (5.55) |
| Infections and infestations | Gastrointestinal fungal infection | 4 | 11.17  (4.17,29.89) | 11.17  (4.17,29.89) | 36.70 | 3.47  (0.58) | 11.08  (4.14) |
| Blood and lymphatic system disorders | Haemorrhagic disorder | 9 | 11.16  (5.79,21.51) | 11.15  (5.79,21.50) | 82.44 | 3.47  (1.55) | 11.06  (5.74) |
| Infections and infestations | Fungaemia | 12 | 11.14  (6.31,19.66) | 11.13  (6.31,19.65) | 109.70 | 3.47  (1.83) | 11.04  (6.25) |
| Infections and infestations | Enterovirus infection | 6 | 11.11  (4.97,24.82) | 11.11  (4.97,24.82) | 54.70 | 3.46  (1.08) | 11.02  (4.93) |
| Neoplasms benign, malignant and unspecified (incl cysts and polyps) | Tumour associated fever | 3 | 11.08  (3.55,34.52) | 11.08  (3.55,34.52) | 27.25 | 3.46  (0.20) | 10.99  (3.52) |
| Gastrointestinal disorders | Oesophageal ulcer haemorrhage | 4 | 11.07  (4.14,29.62) | 11.07  (4.14,29.62) | 36.30 | 3.46  (0.58) | 10.98  (4.10) |
| Blood and lymphatic system disorders | Platelet disorder | 20 | 11.03  (7.10,17.14) | 11.03  (7.10,17.12) | 180.74 | 3.45  (2.26) | 10.94  (7.04) |
| Infections and infestations | Parotitis | 9 | 11.01  (5.71,21.23) | 11.01  (5.71,21.22) | 81.17 | 3.45  (1.54) | 10.92  (5.66) |
| Neoplasms benign, malignant and unspecified (incl cysts and polyps) | Myeloid leukaemia | 4 | 10.90  (4.07,29.16) | 10.90  (4.07,29.15) | 35.63 | 3.43  (0.57) | 10.81  (4.04) |
| Injury, poisoning and procedural complications | Transplantation complication | 3 | 10.85  (3.48,33.80) | 10.85  (3.48,33.80) | 26.58 | 3.43  (0.19) | 10.76  (3.45) |
| Investigations | Klebsiella test positive | 5 | 10.83  (4.49,26.12) | 10.83  (4.49,26.11) | 44.21 | 3.43  (0.85) | 10.74  (4.45) |
| Investigations | Venous pressure jugular increased | 3 | 10.66  (3.42,33.21) | 10.66  (3.42,33.21) | 26.03 | 3.40  (0.19) | 10.58  (3.39) |
| Infections and infestations | Sepsis | 838 | 10.65  (9.95,11.41) | 10.47  (9.79,11.20) | 7131.50 | 3.38  (3.26) | 10.39  (9.70) |
| Metabolism and nutrition disorders | Hyperchloraemia | 5 | 10.50  (4.35,25.31) | 10.49  (4.35,25.31) | 42.58 | 3.38  (0.83) | 10.41  (4.32) |
| Nervous system disorders | Toxic neuropathy | 3 | 10.39  (3.33,32.36) | 10.39  (3.33,32.36) | 25.24 | 3.37  (0.18) | 10.31  (3.31) |
| Congenital, familial and genetic disorders | Cytogenetic abnormality | 10 | 10.34  (5.55,19.26) | 10.33  (5.55,19.26) | 83.61 | 3.36  (1.60) | 10.26  (5.50) |
| Infections and infestations | Staphylococcal bacteraemia | 30 | 10.21  (7.13,14.63) | 10.21  (7.13,14.62) | 247.15 | 3.34  (2.45) | 10.13  (7.07) |
| Infections and infestations | Cellulitis staphylococcal | 5 | 10.13  (4.20,24.43) | 10.13  (4.20,24.42) | 40.81 | 3.33  (0.82) | 10.05  (4.17) |
| Infections and infestations | Klebsiella bacteraemia | 6 | 10.06  (4.51,22.47) | 10.06  (4.51,22.47) | 48.56 | 3.32  (1.03) | 9.99  (4.47) |
| Infections and infestations | Abscess neck | 5 | 10.05  (4.17,24.23) | 10.05  (4.17,24.22) | 40.40 | 3.32  (0.81) | 9.97  (4.14) |
| Infections and infestations | Oesophageal candidiasis | 27 | 9.92  (6.79,14.49) | 9.92  (6.79,14.48) | 214.77 | 3.30  (2.36) | 9.85  (6.74) |
| Blood and lymphatic system disorders | Thrombocytopenia | 768 | 9.91  (9.22,10.64) | 9.75  (9.09,10.46) | 5995.41 | 3.28  (3.15) | 9.68  (9.01) |
| Nervous system disorders | Frontotemporal dementia | 3 | 9.89  (3.17,30.79) | 9.88  (3.17,30.79) | 23.77 | 3.29  (0.16) | 9.81  (3.15) |
| Respiratory, thoracic and mediastinal disorders | Pneumonitis | 180 | 9.81  (8.47,11.37) | 9.78  (8.45,11.32) | 1407.93 | 3.28  (3.00) | 9.71  (8.38) |
| Infections and infestations | Mucormycosis | 21 | 9.78  (6.37,15.03) | 9.78  (6.37,15.02) | 164.22 | 3.28  (2.18) | 9.71  (6.32) |
| Infections and infestations | Septic embolus | 6 | 9.72  (4.35,21.70) | 9.71  (4.35,21.69) | 46.54 | 3.27  (1.01) | 9.65  (4.32) |
| Infections and infestations | Anal abscess | 36 | 9.57  (6.89,13.28) | 9.56  (6.89,13.27) | 273.79 | 3.25  (2.47) | 9.49  (6.84) |
| General disorders and administration site conditions | Administration site pain | 7 | 9.48  (4.51,19.95) | 9.48  (4.51,19.94) | 52.70 | 3.24  (1.17) | 9.42  (4.48) |
| Neoplasms benign, malignant and unspecified (incl cysts and polyps) | Hypergammaglobulinaemia benign monoclonal | 4 | 9.47  (3.54,25.32) | 9.46  (3.54,25.31) | 30.05 | 3.23  (0.51) | 9.40  (3.51) |
| Infections and infestations | Haematoma infection | 4 | 9.43  (3.53,25.22) | 9.43  (3.53,25.22) | 29.91 | 3.23  (0.51) | 9.36  (3.50) |
| Blood and lymphatic system disorders | Myelosuppression | 161 | 9.33  (7.99,10.90) | 9.30  (7.97,10.86) | 1184.11 | 3.21  (2.91) | 9.24  (7.91) |
| General disorders and administration site conditions | Injection site necrosis | 14 | 9.32  (5.51,15.78) | 9.32  (5.51,15.77) | 103.21 | 3.21  (1.83) | 9.26  (5.47) |
| Investigations | Blood bilirubin decreased | 4 | 9.32  (3.49,24.93) | 9.32  (3.49,24.93) | 29.49 | 3.21  (0.51) | 9.26  (3.46) |
| Infections and infestations | Cytomegalovirus infection reactivation | 21 | 9.18  (5.97,14.10) | 9.17  (5.97,14.09) | 151.80 | 3.19  (2.12) | 9.11  (5.93) |
| Gastrointestinal disorders | Gastrointestinal toxicity | 28 | 9.13  (6.29,13.24) | 9.12  (6.29,13.23) | 200.96 | 3.18  (2.29) | 9.06  (6.25) |
| Blood and lymphatic system disorders | Neutropenia | 843 | 9.08  (8.48,9.72) | 8.93  (8.35,9.55) | 5902.74 | 3.15  (3.03) | 8.87  (8.28) |
| Skin and subcutaneous tissue disorders | Panniculitis | 16 | 9.06  (5.54,14.81) | 9.05  (5.54,14.80) | 113.80 | 3.17  (1.91) | 9.00  (5.50) |
| Infections and infestations | Post procedural sepsis | 4 | 8.98  (3.36,24.01) | 8.98  (3.36,24.01) | 28.16 | 3.16  (0.49) | 8.92  (3.34) |
| Gastrointestinal disorders | Enterocolitis haemorrhagic | 10 | 8.97  (4.81,16.71) | 8.97  (4.81,16.70) | 70.27 | 3.16  (1.50) | 8.91  (4.78) |
| Gastrointestinal disorders | Anal ulcer | 5 | 8.95  (3.71,21.57) | 8.95  (3.71,21.56) | 35.04 | 3.15  (0.76) | 8.89  (3.69) |
| Infections and infestations | Pneumonia mycoplasmal | 5 | 8.90  (3.69,21.44) | 8.89  (3.69,21.44) | 34.78 | 3.14  (0.75) | 8.84  (3.67) |
| General disorders and administration site conditions | Fat necrosis | 4 | 8.87  (3.32,23.71) | 8.87  (3.32,23.71) | 27.71 | 3.14  (0.48) | 8.81  (3.29) |
| Renal and urinary disorders | Cystitis haemorrhagic | 25 | 8.84  (5.96,13.10) | 8.83  (5.96,13.09) | 172.37 | 3.13  (2.19) | 8.77  (5.92) |
| Blood and lymphatic system disorders | Bone marrow failure | 136 | 8.78  (7.42,10.40) | 8.76  (7.40,10.36) | 928.16 | 3.12  (2.79) | 8.70  (7.35) |
| Gastrointestinal disorders | Gastritis haemorrhagic | 10 | 8.77  (4.71,16.34) | 8.77  (4.71,16.33) | 68.33 | 3.12  (1.48) | 8.71  (4.68) |
| Neoplasms benign, malignant and unspecified (incl cysts and polyps) | Pyogenic granuloma | 3 | 8.76  (2.81,27.26) | 8.76  (2.81,27.25) | 20.46 | 3.12  (0.12) | 8.70  (2.79) |
| Nervous system disorders | Peripheral sensorimotor neuropathy | 7 | 8.72  (4.15,18.34) | 8.72  (4.15,18.34) | 47.50 | 3.12  (1.12) | 8.66  (4.12) |
| Infections and infestations | Tuberculous pleurisy | 3 | 8.67  (2.79,27.00) | 8.67  (2.79,27.00) | 20.22 | 3.11  (0.12) | 8.62  (2.77) |
| Infections and infestations | Atypical pneumonia | 21 | 8.57  (5.58,13.17) | 8.57  (5.58,13.16) | 139.42 | 3.09  (2.05) | 8.52  (5.54) |
| Investigations | Blast cells present | 3 | 8.45  (2.72,26.31) | 8.45  (2.72,26.31) | 19.58 | 3.07  (0.11) | 8.40  (2.70) |
| Social circumstances | Refusal of treatment by relative | 3 | 8.41  (2.70,26.19) | 8.41  (2.70,26.19) | 19.47 | 3.06  (0.11) | 8.36  (2.69) |
| Infections and infestations | Streptococcal bacteraemia | 6 | 8.34  (3.74,18.61) | 8.34  (3.74,18.61) | 38.49 | 3.05  (0.93) | 8.29  (3.71) |
| Infections and infestations | Cytomegalovirus colitis | 12 | 8.26  (4.68,14.57) | 8.26  (4.68,14.57) | 76.05 | 3.04  (1.60) | 8.21  (4.65) |
| Infections and infestations | Rotavirus infection | 3 | 8.21  (2.64,25.55) | 8.21  (2.64,25.54) | 18.86 | 3.03  (0.10) | 8.16  (2.62) |
| Infections and infestations | Infective exacerbation of chronic obstructive airways disease | 4 | 8.19  (3.06,21.88) | 8.19  (3.06,21.88) | 25.06 | 3.02  (0.45) | 8.14  (3.04) |
| Infections and infestations | Streptococcal sepsis | 7 | 8.14  (3.87,17.12) | 8.14  (3.87,17.12) | 43.55 | 3.02  (1.08) | 8.09  (3.85) |
| General disorders and administration site conditions | Catheter site swelling | 5 | 8.13  (3.37,19.59) | 8.13  (3.37,19.58) | 31.05 | 3.01  (0.71) | 8.08  (3.35) |
| Investigations | Blood bicarbonate increased | 3 | 8.06  (2.59,25.10) | 8.06  (2.59,25.10) | 18.44 | 3.00  (0.09) | 8.02  (2.58) |
| Infections and infestations | Pharyngeal abscess | 3 | 8.06  (2.59,25.10) | 8.06  (2.59,25.10) | 18.44 | 3.00  (0.09) | 8.02  (2.58) |
| Blood and lymphatic system disorders | Splenic haemorrhage | 3 | 7.99  (2.57,24.88) | 7.99  (2.57,24.88) | 18.24 | 2.99  (0.09) | 7.95  (2.55) |
| Infections and infestations | Skin candida | 3 | 7.96  (2.56,24.77) | 7.96  (2.56,24.77) | 18.14 | 2.98  (0.09) | 7.91  (2.54) |
| Musculoskeletal and connective tissue disorders | Necrotising myositis | 4 | 7.88  (2.95,21.07) | 7.88  (2.95,21.07) | 23.88 | 2.97  (0.43) | 7.84  (2.93) |
| Infections and infestations | Brain abscess | 14 | 7.87  (4.65,13.31) | 7.86  (4.65,13.30) | 83.35 | 2.97  (1.68) | 7.82  (4.62) |
| Neoplasms benign, malignant and unspecified (incl cysts and polyps) | Epstein-Barr virus associated lymphoma | 3 | 7.77  (2.50,24.19) | 7.77  (2.50,24.19) | 17.60 | 2.95  (0.08) | 7.73  (2.48) |
| Infections and infestations | Alpha haemolytic streptococcal infection | 5 | 7.76  (3.22,18.69) | 7.76  (3.22,18.69) | 29.25 | 2.95  (0.68) | 7.72  (3.20) |
| Infections and infestations | Epiglottitis | 3 | 7.68  (2.47,23.89) | 7.68  (2.47,23.89) | 17.31 | 2.93  (0.07) | 7.64  (2.45) |
| Skin and subcutaneous tissue disorders | Erythema nodosum | 19 | 7.67  (4.89,12.04) | 7.67  (4.88,12.04) | 109.50 | 2.93  (1.87) | 7.63  (4.86) |
| Infections and infestations | Clostridium difficile colitis | 58 | 7.66  (5.91,9.92) | 7.65  (5.91,9.90) | 333.21 | 2.93  (2.40) | 7.61  (5.88) |
| General disorders and administration site conditions | Catheter site inflammation | 3 | 7.61  (2.45,23.69) | 7.61  (2.45,23.69) | 17.13 | 2.92  (0.07) | 7.57  (2.43) |
| General disorders and administration site conditions | Therapy non-responder | 291 | 7.60  (6.77,8.53) | 7.55  (6.73,8.47) | 1645.70 | 2.91  (2.71) | 7.51  (6.69) |
| Immune system disorders | Engraftment syndrome | 3 | 7.57  (2.43,23.54) | 7.57  (2.43,23.54) | 16.99 | 2.91  (0.07) | 7.53  (2.42) |
| Infections and infestations | Liver abscess | 17 | 7.57  (4.70,12.19) | 7.56  (4.70,12.18) | 96.24 | 2.91  (1.78) | 7.52  (4.67) |
| Infections and infestations | Klebsiella infection | 26 | 7.55  (5.14,11.11) | 7.55  (5.13,11.10) | 146.80 | 2.91  (2.04) | 7.51  (5.11) |
| Infections and infestations | Arthritis bacterial | 22 | 7.52  (4.95,11.44) | 7.52  (4.95,11.43) | 123.61 | 2.90  (1.94) | 7.48  (4.92) |
| Blood and lymphatic system disorders | Disseminated intravascular coagulation | 78 | 7.49  (5.99,9.35) | 7.47  (5.98,9.34) | 434.89 | 2.89  (2.46) | 7.44  (5.95) |
| Infections and infestations | Sialoadenitis | 7 | 7.47  (3.56,15.72) | 7.47  (3.56,15.71) | 39.02 | 2.89  (1.02) | 7.43  (3.54) |
| Infections and infestations | Lung abscess | 11 | 7.45  (4.12,13.49) | 7.45  (4.12,13.48) | 61.09 | 2.89  (1.44) | 7.41  (4.10) |
| Infections and infestations | Parainfluenzae virus infection | 8 | 7.38  (3.68,14.79) | 7.38  (3.68,14.78) | 43.85 | 2.88  (1.14) | 7.34  (3.66) |
| Blood and lymphatic system disorders | Lymphatic disorder | 3 | 7.33  (2.35,22.79) | 7.32  (2.35,22.79) | 16.29 | 2.87  (0.05) | 7.29  (2.34) |
| Investigations | Full blood count decreased | 104 | 7.29  (6.01,8.84) | 7.27  (6.00,8.82) | 559.63 | 2.86  (2.49) | 7.24  (5.97) |
| Respiratory, thoracic and mediastinal disorders | Acute pulmonary oedema | 30 | 7.29  (5.09,10.44) | 7.28  (5.09,10.43) | 161.70 | 2.86  (2.07) | 7.25  (5.06) |
| Infections and infestations | Lymph node tuberculosis | 4 | 7.11  (2.66,18.99) | 7.11  (2.66,18.99) | 20.86 | 2.82  (0.38) | 7.07  (2.65) |
| Blood and lymphatic system disorders | Thrombocytosis | 21 | 7.05  (4.59,10.83) | 7.05  (4.59,10.83) | 108.45 | 2.81  (1.84) | 7.02  (4.57) |
| Gastrointestinal disorders | Subileus | 11 | 7.02  (3.88,12.69) | 7.01  (3.88,12.69) | 56.41 | 2.80  (1.38) | 6.98  (3.86) |
| Injury, poisoning and procedural complications | Subdural haematoma | 73 | 6.90  (5.48,8.69) | 6.89  (5.48,8.67) | 365.80 | 2.78  (2.33) | 6.86  (5.45) |
| Vascular disorders | Aortitis | 3 | 6.87  (2.21,21.38) | 6.87  (2.21,21.38) | 14.97 | 2.77  (0.03) | 6.84  (2.20) |
| Infections and infestations | Haematological infection | 7 | 6.85  (3.26,14.40) | 6.85  (3.26,14.39) | 34.76 | 2.77  (0.96) | 6.82  (3.24) |
| Renal and urinary disorders | Renal vein thrombosis | 3 | 6.80  (2.19,21.14) | 6.80  (2.19,21.14) | 14.75 | 2.76  (0.02) | 6.76  (2.17) |
| Infections and infestations | Hepatic infection | 6 | 6.79  (3.04,15.14) | 6.78  (3.04,15.13) | 29.43 | 2.76  (0.80) | 6.75  (3.03) |
| Cardiac disorders | Cardiopulmonary failure | 20 | 6.78  (4.37,10.52) | 6.78  (4.37,10.51) | 97.93 | 2.75  (1.77) | 6.74  (4.35) |
| Blood and lymphatic system disorders | Leukocytosis | 87 | 6.77  (5.48,8.36) | 6.76  (5.48,8.35) | 424.87 | 2.75  (2.35) | 6.73  (5.45) |
| Investigations | Platelet count decreased | 513 | 6.77  (6.20,7.38) | 6.70  (6.15,7.30) | 2478.12 | 2.74  (2.59) | 6.67  (6.11) |
| Gastrointestinal disorders | Enterocolitis | 25 | 6.69  (4.52,9.92) | 6.69  (4.52,9.91) | 120.32 | 2.74  (1.88) | 6.66  (4.49) |
| Neoplasms benign, malignant and unspecified (incl cysts and polyps) | Monoclonal gammopathy | 3 | 6.67  (2.15,20.76) | 6.67  (2.15,20.76) | 14.39 | 2.73  (0.01) | 6.64  (2.14) |
| Immune system disorders | Chronic graft versus host disease in skin | 4 | 6.65  (2.49,17.77) | 6.65  (2.49,17.77) | 19.11 | 2.73  (0.34) | 6.62  (2.48) |
| Gastrointestinal disorders | Proctitis | 13 | 6.63  (3.84,11.44) | 6.63  (3.84,11.43) | 61.80 | 2.72  (1.46) | 6.60  (3.83) |
| Respiratory, thoracic and mediastinal disorders | Upper respiratory tract inflammation | 6 | 6.58  (2.95,14.69) | 6.58  (2.95,14.69) | 28.26 | 2.71  (0.77) | 6.55  (2.94) |
| Infections and infestations | Pneumonia bacterial | 40 | 6.55  (4.80,8.93) | 6.54  (4.79,8.92) | 186.77 | 2.70  (2.07) | 6.51  (4.77) |
| Infections and infestations | Colonic abscess | 4 | 6.51  (2.44,17.40) | 6.51  (2.44,17.39) | 18.56 | 2.70  (0.33) | 6.48  (2.43) |
| Infections and infestations | Pulmonary nocardiosis | 3 | 6.49  (2.09,20.17) | 6.49  (2.09,20.17) | 13.85 | 2.69  (0.00) | 6.46  (2.08) |
| Injury, poisoning and procedural complications | Splenic rupture | 8 | 6.47  (3.23,12.97) | 6.47  (3.23,12.97) | 36.82 | 2.69  (1.04) | 6.44  (3.22) |
| Blood and lymphatic system disorders | Lymphadenitis | 11 | 6.42  (3.55,11.61) | 6.42  (3.55,11.61) | 50.08 | 2.68  (1.31) | 6.39  (3.53) |
| Injury, poisoning and procedural complications | Subdural haemorrhage | 13 | 6.37  (3.69,10.99) | 6.37  (3.69,10.98) | 58.53 | 2.66  (1.42) | 6.34  (3.68) |
| Gastrointestinal disorders | Anal fissure | 23 | 6.36  (4.22,9.58) | 6.36  (4.22,9.58) | 103.32 | 2.66  (1.78) | 6.33  (4.20) |
| Respiratory, thoracic and mediastinal disorders | Pulmonary haemorrhage | 37 | 6.32  (4.57,8.73) | 6.31  (4.57,8.72) | 164.64 | 2.65  (1.99) | 6.29  (4.55) |
| Infections and infestations | Infection | 620 | 6.31  (5.83,6.83) | 6.23  (5.76,6.74) | 2716.29 | 2.63  (2.50) | 6.21  (5.73) |
| Infections and infestations | Abscess jaw | 5 | 6.25  (2.60,15.06) | 6.25  (2.60,15.06) | 21.96 | 2.64  (0.55) | 6.23  (2.59) |
| Investigations | Haemoglobin abnormal | 27 | 6.11  (4.19,8.92) | 6.11  (4.18,8.91) | 114.75 | 2.60  (1.82) | 6.08  (4.17) |
| Nervous system disorders | Cerebral haemorrhage | 158 | 6.09  (5.21,7.12) | 6.07  (5.19,7.10) | 666.35 | 2.60  (2.32) | 6.05  (5.17) |
| Hepatobiliary disorders | Hyperbilirubinaemia | 44 | 6.08  (4.52,8.18) | 6.08  (4.52,8.17) | 185.67 | 2.60  (2.01) | 6.05  (4.50) |
| Metabolism and nutrition disorders | Cachexia | 23 | 6.08  (4.03,9.16) | 6.07  (4.03,9.15) | 97.02 | 2.60  (1.73) | 6.05  (4.02) |
| Infections and infestations | Pneumonia | 1432 | 6.07  (5.76,6.40) | 5.91  (5.61,6.22) | 5841.83 | 2.56  (2.47) | 5.88  (5.58) |
| Respiratory, thoracic and mediastinal disorders | Pulmonary alveolar haemorrhage | 23 | 6.07  (4.03,9.14) | 6.06  (4.03,9.13) | 96.79 | 2.59  (1.73) | 6.04  (4.01) |
| Cardiac disorders | Cardiac failure acute | 28 | 6.07  (4.18,8.79) | 6.06  (4.18,8.79) | 117.80 | 2.59  (1.83) | 6.04  (4.16) |
| Infections and infestations | Catheter site infection | 16 | 6.04  (3.70,9.87) | 6.04  (3.70,9.87) | 66.96 | 2.59  (1.51) | 6.02  (3.68) |
| Respiratory, thoracic and mediastinal disorders | Lung consolidation | 12 | 5.98  (3.39,10.55) | 5.98  (3.39,10.54) | 49.52 | 2.57  (1.31) | 5.95  (3.38) |
| Infections and infestations | Urinary tract infection bacterial | 11 | 5.97  (3.30,10.81) | 5.97  (3.30,10.80) | 45.33 | 2.57  (1.24) | 5.95  (3.29) |
| Renal and urinary disorders | Urinary tract obstruction | 12 | 5.87  (3.33,10.35) | 5.87  (3.33,10.35) | 48.25 | 2.55  (1.29) | 5.85  (3.32) |
| Investigations | Monocyte count decreased | 5 | 5.86  (2.43,14.11) | 5.86  (2.43,14.11) | 20.05 | 2.54  (0.51) | 5.84  (2.42) |
| Infections and infestations | Perirectal abscess | 5 | 5.84  (2.42,14.05) | 5.84  (2.42,14.05) | 19.95 | 2.54  (0.51) | 5.81  (2.41) |
| Cardiac disorders | Pericardial effusion | 94 | 5.80  (4.74,7.11) | 5.79  (4.73,7.09) | 371.17 | 2.53  (2.16) | 5.77  (4.71) |
| General disorders and administration site conditions | Systemic inflammatory response syndrome | 16 | 5.80  (3.55,9.47) | 5.80  (3.55,9.47) | 63.19 | 2.53  (1.47) | 5.77  (3.53) |
| Gastrointestinal disorders | Diverticular perforation | 9 | 5.78  (3.00,11.12) | 5.78  (3.00,11.12) | 35.37 | 2.52  (1.05) | 5.75  (2.99) |
| Investigations | Escherichia test positive | 5 | 5.77  (2.40,13.90) | 5.77  (2.40,13.89) | 19.63 | 2.52  (0.50) | 5.75  (2.39) |
| Surgical and medical procedures | Platelet transfusion | 5 | 5.72  (2.38,13.78) | 5.72  (2.38,13.78) | 19.39 | 2.51  (0.49) | 5.70  (2.37) |
| Neoplasms benign, malignant and unspecified (incl cysts and polyps) | Acute lymphocytic leukaemia | 12 | 5.72  (3.24,10.08) | 5.72  (3.24,10.08) | 46.48 | 2.51  (1.26) | 5.69  (3.23) |
| Gastrointestinal disorders | Colitis ischaemic | 27 | 5.71  (3.91,8.33) | 5.71  (3.91,8.33) | 104.34 | 2.51  (1.74) | 5.69  (3.89) |
| Infections and infestations | Atypical mycobacterial infection | 5 | 5.66  (2.35,13.64) | 5.66  (2.35,13.64) | 19.12 | 2.50  (0.49) | 5.64  (2.34) |
| Infections and infestations | JC virus infection | 6 | 5.66  (2.54,12.62) | 5.66  (2.54,12.61) | 22.90 | 2.49  (0.67) | 5.64  (2.53) |
| Infections and infestations | Myelitis | 6 | 5.65  (2.53,12.61) | 5.65  (2.53,12.61) | 22.87 | 2.49  (0.67) | 5.63  (2.53) |
| Endocrine disorders | Thyrotoxic crisis | 4 | 5.64  (2.11,15.05) | 5.64  (2.11,15.05) | 15.18 | 2.49  (0.25) | 5.61  (2.10) |
| General disorders and administration site conditions | Physical deconditioning | 8 | 5.61  (2.80,11.24) | 5.61  (2.80,11.24) | 30.19 | 2.48  (0.92) | 5.59  (2.79) |
| Blood and lymphatic system disorders | White blood cell disorder | 11 | 5.60  (3.10,10.14) | 5.60  (3.10,10.13) | 41.42 | 2.48  (1.18) | 5.58  (3.09) |
| Hepatobiliary disorders | Congestive hepatopathy | 6 | 5.60  (2.51,12.49) | 5.60  (2.51,12.49) | 22.58 | 2.48  (0.66) | 5.58  (2.50) |
| Respiratory, thoracic and mediastinal disorders | Acute lung injury | 5 | 5.56  (2.31,13.38) | 5.56  (2.31,13.38) | 18.60 | 2.47  (0.47) | 5.54  (2.30) |
| Infections and infestations | H1N1 influenza | 6 | 5.55  (2.49,12.38) | 5.55  (2.49,12.38) | 22.28 | 2.47  (0.65) | 5.53  (2.48) |
| Infections and infestations | Abdominal infection | 10 | 5.55  (2.98,10.33) | 5.55  (2.98,10.32) | 37.10 | 2.47  (1.10) | 5.53  (2.97) |
| Investigations | Blood culture positive | 13 | 5.52  (3.20,9.52) | 5.52  (3.20,9.52) | 47.92 | 2.46  (1.28) | 5.50  (3.19) |
| Vascular disorders | Phlebitis | 21 | 5.51  (3.59,8.46) | 5.51  (3.59,8.45) | 77.10 | 2.46  (1.57) | 5.49  (3.57) |
| Cardiac disorders | Aortic valve stenosis | 7 | 5.49  (2.61,11.54) | 5.49  (2.61,11.53) | 25.59 | 2.45  (0.79) | 5.47  (2.60) |
| Infections and infestations | Erysipelas | 20 | 5.47  (3.52,8.49) | 5.47  (3.52,8.48) | 72.68 | 2.45  (1.54) | 5.45  (3.51) |
| Vascular disorders | Venoocclusive disease | 10 | 5.42  (2.91,10.08) | 5.42  (2.91,10.08) | 35.85 | 2.43  (1.07) | 5.40  (2.90) |
| Nervous system disorders | Haemorrhage intracranial | 61 | 5.40  (4.19,6.94) | 5.39  (4.19,6.93) | 217.15 | 2.42  (1.96) | 5.37  (4.18) |
| Infections and infestations | Orchitis | 4 | 5.37  (2.01,14.34) | 5.37  (2.01,14.34) | 14.16 | 2.42  (0.22) | 5.35  (2.00) |
| General disorders and administration site conditions | Multiple organ dysfunction syndrome | 172 | 5.36  (4.62,6.23) | 5.35  (4.60,6.21) | 605.72 | 2.41  (2.16) | 5.33  (4.59) |
| Respiratory, thoracic and mediastinal disorders | Obliterative bronchiolitis | 5 | 5.30  (2.20,12.76) | 5.30  (2.20,12.76) | 17.38 | 2.40  (0.44) | 5.28  (2.19) |
| Infections and infestations | Injection site cellulitis | 7 | 5.28  (2.51,11.10) | 5.28  (2.51,11.09) | 24.18 | 2.40  (0.76) | 5.26  (2.50) |
| Infections and infestations | Rhinovirus infection | 12 | 5.28  (2.99,9.31) | 5.28  (2.99,9.31) | 41.45 | 2.40  (1.18) | 5.26  (2.98) |
| Immune system disorders | Haemophagocytic lymphohistiocytosis | 34 | 5.20  (3.72,7.29) | 5.20  (3.71,7.28) | 114.92 | 2.37  (1.72) | 5.18  (3.70) |
| Investigations | Troponin I increased | 6 | 5.14  (2.31,11.47) | 5.14  (2.31,11.47) | 19.94 | 2.36  (0.60) | 5.13  (2.30) |
| Cardiac disorders | Left ventricular failure | 13 | 5.10  (2.96,8.80) | 5.10  (2.96,8.79) | 42.68 | 2.35  (1.20) | 5.08  (2.95) |
| Respiratory, thoracic and mediastinal disorders | Lung infiltration | 35 | 5.09  (3.65,7.09) | 5.08  (3.65,7.08) | 114.37 | 2.34  (1.70) | 5.07  (3.64) |
| Investigations | White blood cell count decreased | 395 | 5.08  (4.60,5.61) | 5.04  (4.57,5.57) | 1277.99 | 2.33  (2.17) | 5.03  (4.55) |
| Skin and subcutaneous tissue disorders | Vascular purpura | 5 | 5.05  (2.10,12.15) | 5.05  (2.10,12.15) | 16.17 | 2.33  (0.41) | 5.03  (2.09) |
| Metabolism and nutrition disorders | Hypoalbuminaemia | 27 | 5.05  (3.46,7.36) | 5.04  (3.46,7.36) | 87.17 | 2.33  (1.59) | 5.03  (3.44) |
| Neoplasms benign, malignant and unspecified (incl cysts and polyps) | Chronic myeloid leukaemia | 17 | 5.04  (3.13,8.12) | 5.04  (3.13,8.11) | 54.80 | 2.33  (1.36) | 5.02  (3.12) |
| Skin and subcutaneous tissue disorders | Skin necrosis | 19 | 5.04  (3.21,7.91) | 5.04  (3.21,7.90) | 61.24 | 2.33  (1.42) | 5.02  (3.20) |
| Infections and infestations | Pseudomonas infection | 29 | 5.01  (3.48,7.22) | 5.01  (3.48,7.21) | 92.63 | 2.32  (1.61) | 4.99  (3.47) |
| Vascular disorders | Venous thrombosis | 15 | 5.01  (3.02,8.32) | 5.01  (3.02,8.32) | 47.93 | 2.32  (1.27) | 4.99  (3.01) |
| Ear and labyrinth disorders | Sudden hearing loss | 7 | 5.00  (2.38,10.50) | 5.00  (2.38,10.50) | 22.29 | 2.32  (0.71) | 4.98  (2.37) |
| Blood and lymphatic system disorders | Anaemia | 685 | 4.99  (4.63,5.38) | 4.93  (4.57,5.31) | 2142.61 | 2.30  (2.18) | 4.91  (4.55) |
| Metabolism and nutrition disorders | Hyperphosphataemia | 7 | 4.97  (2.37,10.44) | 4.97  (2.37,10.44) | 22.12 | 2.31  (0.71) | 4.95  (2.36) |
| Infections and infestations | Escherichia infection | 28 | 4.96  (3.42,7.19) | 4.96  (3.42,7.19) | 88.15 | 2.31  (1.58) | 4.94  (3.41) |
| Cardiac disorders | Cardiac tamponade | 17 | 4.92  (3.06,7.93) | 4.92  (3.06,7.92) | 52.89 | 2.29  (1.33) | 4.90  (3.05) |
| Skin and subcutaneous tissue disorders | Haemorrhage subcutaneous | 13 | 4.90  (2.84,8.45) | 4.90  (2.84,8.44) | 40.16 | 2.29  (1.16) | 4.88  (2.83) |
| Infections and infestations | Endocarditis | 18 | 4.89  (3.08,7.77) | 4.89  (3.08,7.77) | 55.52 | 2.29  (1.35) | 4.88  (3.07) |
| Musculoskeletal and connective tissue disorders | Chondrocalcinosis | 5 | 4.86  (2.02,11.71) | 4.86  (2.02,11.71) | 15.29 | 2.28  (0.38) | 4.85  (2.01) |
| Infections and infestations | COVID-19 pneumonia | 41 | 4.85  (3.57,6.60) | 4.85  (3.57,6.59) | 124.83 | 2.27  (1.70) | 4.83  (3.56) |
| Infections and infestations | Meningitis bacterial | 5 | 4.84  (2.01,11.64) | 4.84  (2.01,11.64) | 15.16 | 2.27  (0.38) | 4.82  (2.00) |
| Infections and infestations | Pneumonia pseudomonal | 7 | 4.78  (2.28,10.04) | 4.78  (2.28,10.04) | 20.84 | 2.25  (0.67) | 4.77  (2.27) |
| Metabolism and nutrition disorders | Hyperuricaemia | 14 | 4.77  (2.82,8.07) | 4.77  (2.82,8.07) | 41.60 | 2.25  (1.18) | 4.76  (2.82) |
| Cardiac disorders | Cardiac dysfunction | 11 | 4.77  (2.64,8.62) | 4.77  (2.64,8.62) | 32.63 | 2.25  (1.02) | 4.75  (2.63) |
| Renal and urinary disorders | Renal tubular acidosis | 7 | 4.75  (2.26,9.99) | 4.75  (2.26,9.98) | 20.67 | 2.24  (0.67) | 4.74  (2.26) |
| Blood and lymphatic system disorders | Haemolysis | 26 | 4.68  (3.18,6.88) | 4.68  (3.18,6.88) | 74.95 | 2.22  (1.48) | 4.67  (3.17) |
| Respiratory, thoracic and mediastinal disorders | Respiratory failure | 247 | 4.68  (4.13,5.30) | 4.66  (4.11,5.27) | 707.41 | 2.22  (2.01) | 4.64  (4.10) |
| Blood and lymphatic system disorders | Bone marrow disorder | 10 | 4.63  (2.49,8.62) | 4.63  (2.49,8.61) | 28.34 | 2.21  (0.92) | 4.62  (2.48) |
| Infections and infestations | Intervertebral discitis | 6 | 4.63  (2.08,10.32) | 4.63  (2.08,10.32) | 17.00 | 2.21  (0.51) | 4.61  (2.07) |
| Gastrointestinal disorders | Large intestine perforation | 23 | 4.61  (3.06,6.94) | 4.61  (3.06,6.94) | 64.69 | 2.20  (1.41) | 4.59  (3.05) |
| Blood and lymphatic system disorders | Splenomegaly | 39 | 4.55  (3.32,6.23) | 4.55  (3.32,6.22) | 107.47 | 2.18  (1.60) | 4.53  (3.31) |
| Nervous system disorders | Subarachnoid haemorrhage | 34 | 4.54  (3.24,6.37) | 4.54  (3.24,6.36) | 93.59 | 2.18  (1.55) | 4.53  (3.23) |
| Blood and lymphatic system disorders | Haemorrhagic diathesis | 12 | 4.51  (2.56,7.96) | 4.51  (2.56,7.95) | 32.70 | 2.17  (1.02) | 4.50  (2.55) |
| Skin and subcutaneous tissue disorders | Cutaneous vasculitis | 15 | 4.43  (2.67,7.35) | 4.42  (2.66,7.34) | 39.61 | 2.14  (1.14) | 4.41  (2.66) |
| Skin and subcutaneous tissue disorders | Hypersensitivity vasculitis | 9 | 4.38  (2.28,8.44) | 4.38  (2.28,8.43) | 23.42 | 2.13  (0.79) | 4.37  (2.27) |
| Infections and infestations | Skin infection | 34 | 4.37  (3.12,6.12) | 4.37  (3.12,6.11) | 87.92 | 2.12  (1.50) | 4.35  (3.11) |
| Musculoskeletal and connective tissue disorders | Myositis | 26 | 4.30  (2.92,6.32) | 4.30  (2.92,6.31) | 65.56 | 2.10  (1.38) | 4.29  (2.92) |
| Gastrointestinal disorders | Colitis | 111 | 4.28  (3.55,5.16) | 4.28  (3.55,5.15) | 277.73 | 2.09  (1.78) | 4.26  (3.54) |
| Infections and infestations | Cellulitis | 158 | 4.28  (3.66,5.01) | 4.27  (3.65,4.99) | 394.55 | 2.09  (1.83) | 4.26  (3.64) |
| General disorders and administration site conditions | Disease progression | 354 | 4.28  (3.85,4.75) | 4.25  (3.83,4.71) | 878.17 | 2.08  (1.92) | 4.24  (3.82) |
| Infections and infestations | Device related sepsis | 7 | 4.27  (2.03,8.97) | 4.27  (2.03,8.97) | 17.46 | 2.09  (0.57) | 4.26  (2.03) |
| Infections and infestations | Injection site infection | 10 | 4.22  (2.27,7.86) | 4.22  (2.27,7.86) | 24.52 | 2.07  (0.83) | 4.21  (2.26) |
| Cardiac disorders | Atrial flutter | 25 | 4.22  (2.85,6.25) | 4.22  (2.85,6.25) | 61.23 | 2.07  (1.34) | 4.21  (2.84) |
| Infections and infestations | Vascular device infection | 13 | 4.17  (2.42,7.19) | 4.17  (2.42,7.19) | 31.21 | 2.06  (0.99) | 4.16  (2.41) |
| Vascular disorders | Thrombophlebitis | 11 | 4.15  (2.30,7.51) | 4.15  (2.30,7.51) | 26.25 | 2.05  (0.88) | 4.14  (2.29) |
| Cardiac disorders | Supraventricular tachycardia | 29 | 4.13  (2.87,5.94) | 4.12  (2.86,5.94) | 68.41 | 2.04  (1.37) | 4.11  (2.86) |
| Gastrointestinal disorders | Intestinal ischaemia | 16 | 4.10  (2.51,6.70) | 4.10  (2.51,6.70) | 37.37 | 2.03  (1.09) | 4.09  (2.50) |
| Blood and lymphatic system disorders | Blood disorder | 24 | 4.10  (2.75,6.12) | 4.10  (2.74,6.12) | 56.01 | 2.03  (1.28) | 4.09  (2.74) |
| Hepatobiliary disorders | Venoocclusive liver disease | 14 | 4.08  (2.41,6.89) | 4.08  (2.41,6.89) | 32.41 | 2.02  (1.01) | 4.07  (2.41) |
| Infections and infestations | Urosepsis | 27 | 4.08  (2.79,5.95) | 4.07  (2.79,5.94) | 62.43 | 2.02  (1.33) | 4.06  (2.78) |
| Vascular disorders | Embolism | 25 | 4.07  (2.75,6.03) | 4.07  (2.75,6.02) | 57.67 | 2.02  (1.29) | 4.06  (2.74) |
| Investigations | Neutrophil count abnormal | 8 | 4.07  (2.03,8.14) | 4.07  (2.03,8.14) | 18.44 | 2.02  (0.63) | 4.06  (2.03) |
| Respiratory, thoracic and mediastinal disorders | Atelectasis | 29 | 4.01  (2.79,5.78) | 4.01  (2.79,5.77) | 65.33 | 2.00  (1.33) | 4.00  (2.78) |
| Infections and infestations | Pneumocystis jirovecii pneumonia | 35 | 4.01  (2.88,5.59) | 4.01  (2.88,5.59) | 78.80 | 2.00  (1.40) | 4.00  (2.87) |
| Investigations | Serum ferritin increased | 15 | 3.98  (2.39,6.60) | 3.97  (2.39,6.60) | 33.29 | 1.99  (1.02) | 3.96  (2.39) |
| Metabolism and nutrition disorders | Hypernatraemia | 14 | 3.94  (2.33,6.67) | 3.94  (2.33,6.66) | 30.66 | 1.98  (0.97) | 3.93  (2.33) |
| Gastrointestinal disorders | Lower gastrointestinal haemorrhage | 22 | 3.92  (2.58,5.96) | 3.92  (2.58,5.95) | 47.66 | 1.97  (1.19) | 3.91  (2.57) |
| Respiratory, thoracic and mediastinal disorders | Acute respiratory distress syndrome | 50 | 3.91  (2.96,5.16) | 3.91  (2.96,5.16) | 107.85 | 1.96  (1.48) | 3.90  (2.95) |
| Gastrointestinal disorders | Ileus | 33 | 3.85  (2.74,5.42) | 3.85  (2.74,5.42) | 69.42 | 1.94  (1.33) | 3.84  (2.73) |
| Infections and infestations | Respiratory tract infection | 68 | 3.83  (3.02,4.86) | 3.82  (3.01,4.85) | 141.42 | 1.93  (1.53) | 3.82  (3.01) |
| Respiratory, thoracic and mediastinal disorders | Acute respiratory failure | 51 | 3.82  (2.90,5.03) | 3.82  (2.90,5.03) | 105.87 | 1.93  (1.45) | 3.81  (2.89) |
| Respiratory, thoracic and mediastinal disorders | Hypoxia | 94 | 3.81  (3.11,4.67) | 3.81  (3.11,4.66) | 193.93 | 1.92  (1.59) | 3.80  (3.10) |
| Gastrointestinal disorders | Ileus paralytic | 12 | 3.81  (2.16,6.71) | 3.81  (2.16,6.71) | 24.77 | 1.93  (0.84) | 3.80  (2.16) |
| Infections and infestations | Bacterial infection | 49 | 3.79  (2.87,5.02) | 3.79  (2.87,5.02) | 100.44 | 1.92  (1.43) | 3.78  (2.86) |
| Nervous system disorders | Haemorrhagic stroke | 22 | 3.78  (2.49,5.75) | 3.78  (2.49,5.75) | 44.90 | 1.92  (1.15) | 3.77  (2.48) |
| Investigations | Blood urea increased | 48 | 3.76  (2.83,4.99) | 3.76  (2.83,4.99) | 96.87 | 1.91  (1.41) | 3.75  (2.82) |
| Infections and infestations | Clostridial infection | 10 | 3.75  (2.02,6.97) | 3.75  (2.02,6.97) | 20.09 | 1.90  (0.71) | 3.74  (2.01) |
| Respiratory, thoracic and mediastinal disorders | Interstitial lung disease | 124 | 3.70  (3.10,4.41) | 3.69  (3.10,4.40) | 242.71 | 1.88  (1.59) | 3.68  (3.09) |
| Infections and infestations | Subcutaneous abscess | 13 | 3.69  (2.14,6.36) | 3.69  (2.14,6.36) | 25.43 | 1.88  (0.86) | 3.68  (2.14) |
| Respiratory, thoracic and mediastinal disorders | Pleural effusion | 164 | 3.68  (3.16,4.30) | 3.67  (3.15,4.28) | 318.52 | 1.87  (1.63) | 3.67  (3.14) |
| Hepatobiliary disorders | Cholangitis | 15 | 3.68  (2.22,6.11) | 3.68  (2.22,6.10) | 29.16 | 1.88  (0.93) | 3.67  (2.21) |
| Gastrointestinal disorders | Anal fistula | 14 | 3.65  (2.16,6.16) | 3.65  (2.16,6.16) | 26.81 | 1.86  (0.88) | 3.64  (2.15) |
| Skin and subcutaneous tissue disorders | Skin reaction | 36 | 3.64  (2.63,5.05) | 3.64  (2.63,5.05) | 68.76 | 1.86  (1.29) | 3.63  (2.62) |
| Blood and lymphatic system disorders | Leukopenia | 125 | 3.53  (2.96,4.21) | 3.53  (2.96,4.20) | 225.65 | 1.81  (1.53) | 3.52  (2.95) |
| Cardiac disorders | Cardiovascular disorder | 68 | 3.50  (2.76,4.44) | 3.50  (2.76,4.44) | 121.02 | 1.80  (1.40) | 3.49  (2.75) |
| Investigations | Blood lactate dehydrogenase increased | 42 | 3.47  (2.56,4.69) | 3.46  (2.56,4.69) | 73.39 | 1.79  (1.27) | 3.46  (2.55) |
| Blood and lymphatic system disorders | Agranulocytosis | 44 | 3.46  (2.57,4.65) | 3.46  (2.57,4.65) | 76.74 | 1.79  (1.28) | 3.45  (2.57) |
| General disorders and administration site conditions | Mucosal inflammation | 64 | 3.46  (2.70,4.42) | 3.45  (2.70,4.41) | 111.33 | 1.79  (1.37) | 3.45  (2.70) |
| Neoplasms benign, malignant and unspecified (incl cysts and polyps) | Diffuse large B-cell lymphoma | 17 | 3.46  (2.15,5.57) | 3.46  (2.15,5.56) | 29.59 | 1.79  (0.92) | 3.45  (2.14) |
| Gastrointestinal disorders | Proctalgia | 16 | 3.43  (2.10,5.60) | 3.43  (2.10,5.60) | 27.46 | 1.77  (0.88) | 3.42  (2.10) |
| Infections and infestations | Device related infection | 42 | 3.43  (2.53,4.64) | 3.43  (2.53,4.64) | 72.02 | 1.77  (1.25) | 3.42  (2.53) |
| Blood and lymphatic system disorders | Granulocytopenia | 15 | 3.42  (2.06,5.68) | 3.42  (2.06,5.68) | 25.64 | 1.77  (0.85) | 3.42  (2.06) |
| Investigations | C-reactive protein increased | 85 | 3.40  (2.75,4.20) | 3.39  (2.74,4.20) | 143.12 | 1.76  (1.41) | 3.39  (2.74) |
| General disorders and administration site conditions | Pyrexia | 819 | 3.33  (3.10,3.56) | 3.28  (3.07,3.51) | 1304.51 | 1.71  (1.61) | 3.28  (3.06) |
| Cardiac disorders | Cardiac failure | 190 | 3.29  (2.85,3.79) | 3.28  (2.84,3.78) | 300.35 | 1.71  (1.48) | 3.27  (2.84) |
| General disorders and administration site conditions | Death | 1943 | 3.28  (3.13,3.43) | 3.18  (3.04,3.32) | 2932.82 | 1.67  (1.60) | 3.17  (3.03) |
| Metabolism and nutrition disorders | Acidosis | 18 | 3.23  (2.03,5.13) | 3.23  (2.03,5.13) | 27.60 | 1.69  (0.87) | 3.22  (2.03) |
| General disorders and administration site conditions | General physical health deterioration | 245 | 3.17  (2.80,3.60) | 3.16  (2.79,3.58) | 361.97 | 1.66  (1.46) | 3.16  (2.78) |
| Skin and subcutaneous tissue disorders | Petechiae | 23 | 3.07  (2.04,4.62) | 3.07  (2.04,4.62) | 31.94 | 1.61  (0.90) | 3.06  (2.03) |
| Investigations | Blood bilirubin increased | 60 | 3.00  (2.33,3.87) | 3.00  (2.33,3.86) | 79.80 | 1.58  (1.17) | 2.99  (2.32) |
| Immune system disorders | Immune system disorder | 28 | 2.98  (2.06,4.32) | 2.98  (2.06,4.32) | 36.78 | 1.57  (0.94) | 2.98  (2.05) |
| Investigations | Red blood cell count decreased | 61 | 2.93  (2.28,3.77) | 2.93  (2.28,3.76) | 77.22 | 1.55  (1.14) | 2.92  (2.27) |
| Infections and infestations | Diverticulitis | 57 | 2.92  (2.25,3.79) | 2.92  (2.25,3.79) | 71.80 | 1.54  (1.12) | 2.92  (2.25) |
| Investigations | Haemoglobin decreased | 215 | 2.85  (2.50,3.26) | 2.84  (2.49,3.25) | 257.00 | 1.51  (1.30) | 2.84  (2.48) |
| Infections and infestations | Fungal infection | 63 | 2.63  (2.06,3.37) | 2.63  (2.05,3.37) | 63.59 | 1.39  (1.00) | 2.63  (2.05) |
| Respiratory, thoracic and mediastinal disorders | Respiratory disorder | 56 | 2.62  (2.02,3.41) | 2.62  (2.02,3.41) | 56.06 | 1.39  (0.96) | 2.62  (2.01) |
| General disorders and administration site conditions | Injection site reaction | 126 | 2.59  (2.18,3.09) | 2.59  (2.17,3.08) | 122.62 | 1.37  (1.10) | 2.58  (2.17) |
| General disorders and administration site conditions | Treatment failure | 139 | 2.54  (2.15,3.00) | 2.53  (2.14,2.99) | 128.67 | 1.34  (1.08) | 2.53  (2.14) |

Note1:ranked by EBGM

Note2:Signals are detected when all the following criteria are met:a ≥ 3, PRR ≥2 and Chi-Square ≥ 4, lower limit of 95% CI of ROR > 1, IC025 > 0, EBGM05 > 2.
